# Sensing the Underground – Ultrastructure and Function of Sensory Organs in Root-Feeding *Melolontha melolontha* (Coleoptera: Scarabaeinae) Larvae

**DOI:** 10.1371/journal.pone.0041357

**Published:** 2012-07-25

**Authors:** Elisabeth J. Eilers, Giovanni Talarico, Bill S. Hansson, Monika Hilker, Andreas Reinecke

**Affiliations:** 1 Freie Universität Berlin, Department of Applied Zoology/Animal Ecology, Berlin, Germany; 2 Max-Planck-Institute for Chemical Ecology, Department of Evolutionary Neuroethology, Jena, Germany; AgroParisTech, France

## Abstract

**Introduction:**

Below ground orientation in insects relies mainly on olfaction and taste. The economic impact of plant root feeding scarab beetle larvae gave rise to numerous phylogenetic and ecological studies. Detailed knowledge of the sensory capacities of these larvae is nevertheless lacking. Here, we present an atlas of the sensory organs on larval head appendages of *Melolontha melolontha*. Our ultrastructural and electrophysiological investigations allow annotation of functions to various sensory structures.

**Results:**

Three out of 17 ascertained sensillum types have olfactory, and 7 gustatory function. These sensillum types are unevenly distributed between antennae and palps. The most prominent chemosensory organs are antennal pore plates that in total are innervated by approximately one thousand olfactory sensory neurons grouped into functional units of three-to-four. In contrast, only two olfactory sensory neurons innervate one sensillum basiconicum on each of the palps. Gustatory sensilla chaetica dominate the apices of all head appendages, while only the palps bear thermo-/hygroreceptors. Electrophysiological responses to CO_2_, an attractant for many root feeders, are exclusively observed in the antennae. Out of 54 relevant volatile compounds, various alcohols, acids, amines, esters, aldehydes, ketones and monoterpenes elicit responses in antennae and palps. All head appendages are characterized by distinct olfactory response profiles that are even enantiomer specific for some compounds.

**Conclusions:**

Chemosensory capacities in *M. melolontha* larvae are as highly developed as in many adult insects. We interpret the functional sensory units underneath the antennal pore plates as cryptic sensilla placodea and suggest that these perceive a broad range of secondary plant metabolites together with CO_2_. Responses to olfactory stimulation of the labial and maxillary palps indicate that typical contact chemo-sensilla have a dual gustatory and olfactory function.

## Introduction

Below ground interactions between plants and herbivores have gained increased attention over the past years (e.g. [1], [2]). Little knowledge is, however, available regarding how rhizophagous herbivores such as scarab beetle larvae locate host roots. In the absence of visual stimuli, olfaction and taste are the core sensory modalities to orient below ground. Sensory head appendages of rhizophagous larvae have been described from phylogenetic perspectives in scarab beetles [3], or studied from a functional point of view in other model or pest organisms [4], [5], [6]. Despite the presence of many pest species within the superfamily Scarabaeoidea, comprising 25,000-to-35,000 species in 8-to-14 families [3], [7], [8], [9], a comprehensive inventory of sensory organs on larval antennae, labial, and maxillary palps is missing. The scarcity of data becomes even more apparent when searching for studies linking morphology, physiology and ecology of insect larvae in general and scarab larvae in particular.

Out of ten basic sensillum types that have been described in adult insects, all except the sensilla squamiformia have also been found in insect larvae [10]. Common sensory structures among coleopteran and lepidopteran larvae are placoid structures on apical antennal segments [11] and maxillary palps [12], digitiform organs on maxillary palps (e.g. [13], [14]) and peg-like sensilla on apices of antennae and palps (e.g. [15], [16]) (cp. Table S1). The conjoint occurrence in various coleopteran and lepidopteran taxa of a broad geographical range, diverse habitats and diets, indicates a highly conserved nature of these structures. Between taxa they differ in number, size and location on head appendages.

Pore plates on larval antennae with hypothesized olfactory function have been demonstrated in Carabidae [11]. Similar structures have olfactory function in adult scarab [17] and Dynastidae beetles [18]. Furthermore, peg-like sensilla of unknown function have been identified on apices of antennae [19], labial and maxillary palps [20] in Scarabaeidae and other Coleoptera (see Table S1). Finally, digitiform organs have been described in larvae of Carabidae [21], Chrysomelidae [22], Curculionidae [23] and Elateridae [15] (Table S1). The putative function of the digitiform organ is hygro-/thermo- [13], or CO_2_-reception [14], and in lepidopteran larvae mechanoreception [24]. Most reference studies, however, are purely descriptive, lacking physiological and ultrastructural investigations of sensory function and organization.

In our model insect *Melolontha melolontha* (L., 1758) (Scarabaeidae: Melolonthinae) it has been postulated that CO_2_ is the only or main attractant below ground [25], [26]. However, CO_2_ receptive structures have not been identified yet [26]. In wireworm larvae, CO_2_ receptive sensilla are suspected to be located on both palpal apices [15]. Recent findings indicate that other compounds of the rhizosphere contribute to orientation or interact with CO_2_ in *Melolontha* larvae [27]. In addition to CO_2_, which is an ubiquitous gas produced by respiring roots and other soil (micro)organisms, plant roots release various water-soluble substances into the soil, such as sugars, organic acids, and amino acids (reviews by [28], [29], [30] and references therein). Gustatory discrimination of food sources based on sugars, amino acids, and isoflavonoids has been shown in rhizophagous clover root weevil and scarab larvae [31], [32]. Volatile compounds are secreted in comparatively limited diversity and quantity from plant roots [33]. However, these compounds act as attractants or deterrents in various scarab larvae [34], [35].

In this study we establish a comprehensive inventory of the sensory structures on the head appendages of *M. melolontha* larvae by scanning and transmission electron microscopy. We present a functional interpretation of our ultrastructural data and an assessment of olfactory responses to compounds known to be behaviorally active in soil dwelling insects, to be present in the rhizosphere of potential host plants, or to structural analogues of these compounds.

## Materials and Methods

### Animals

*Melolontha melolontha* (Linnaeus, 1758) larvae were collected in May 2010 and April 2011 from a meadow in Hessenthal, Bavaria, Germany (49°93′ N, 9°26′O). Larvae were kept individually in small pots filled with clay substrate (Klasmann-Deilmann GmbH, Geeste, Germany) in a climate chamber under dark conditions at 14°C and 70% humidity and fed carrots *ad libitum*. Third instar larvae were used in all experiments. Collected second instar larvae were allowed to molt before use.

### Scanning electron microscopy (SEM)

After rinsing with tap water, five specimens were decapitated, and the heads were submerged in Sörensen phosphate buffer (0.1M, pH 7.2, 1.8% sucrose) before antennae, labial and maxillary palps were removed and placed in 50% ethanol. Samples were dehydrated in ethanol (EtOH) (60, 70, 80% each step twice for 10 minutes; 90%, 96% for 10 minutes each, absolute EtOH overnight). Subsequently, the specimens were critical point-dried using a BAL-TEC CPD 030, mounted on aluminium stubs with adhesive film, and sputter coated with gold on a BAL-TEC SCD005 prior examination with a LEO 1450 VP scanning electron microscope.

### Transmission electron microscopy (TEM)

After rinsing and decapitation, antennae and palps from two specimens were dissected in chilled Sörensen phosphate buffer (0.1M, pH 7.2, 1,8% sucrose). Antennae were divided into antennal tip, rest of the first apical segment, and proximal half of post-apical segment; tips of palps and cylinder of apical segment of maxillary palps were dissected. Samples were fixed for 12 hours with 2.5% glutaraldehyde in phosphate buffer at 4°C. Samples were rinsed two times for 10 minutes with chilled phosphate buffer before the buffer was replaced by 2% phosphate buffered osmium tetroxide and stored for 12 hours at 4°C. After rinsing three times for 10 minutes with chilled phosphate buffer, the samples were dehydrated in EtOH in ascending concentrations (see above). Dehydrated samples were embedded in Spurŕs resin [36] and polymerized for 24 hours at 65°C. Ultrathin sections (50–70 nm) were cut with a Diatome diamond knife (Ultra 35°) on a Reichert Ultracut microtome. Sections were collected on Pioloform®-coated mesh or single slot copper grids and examined without additional staining with a Zeiss CEM 902A (with a TVIPS FastScan camera) or a JEOL JEM 1011 (with a Olympus Megaview III camera) transmission electron microscope.

### Electroantennograms (EAGs) and electropalpograms (EPGs)

White grubs were fixed in slit silicone tubes (ca. 2cm long ID = 6mm) supported by a bandage of Parafilm (Pechiney Plastic Packaging), leaving the head appendages and hindmost part of the abdomen free. Microcapillary glass electrodes (tip OD ca. 3µm) with Ringeŕs solution and a silver wire provided electrical contact via a Syntech 10× universal probe pre-amplifier (Ockenfels SYNTECH GmbH, Kirchzarten, Germany) to a Syntech IDAC 4 D/A-converter. The indifferent electrode was inserted into the larval abdomen [37]. The measuring electrode was positioned laterally on the apical segment of the respective head appendage without penetration of the cuticle. Sensilla on the tip of all appendages, antennal pore plates and the digitiform organ on the maxillary palps were not covered by the electrode. Signals were recorded on a PC using Syntech EAG Software with 50/60Hz electric noise suppression and the ‘EAG-filter’ activated. Larval head appendages were subjected to a constant flow (1 L/min) of charcoal-filtered, humidified air through a stainless steel tube (ID 8mm) terminating 1cm from the preparation and with two lateral holes (2 mm ID) about 1 cm upstream of the outlet. Stimuli were applied by puffing charcoal filtered air (500mL/min, 0.5 s per stimulus, 4mL in total) through Pasteur-pipettes with odor-laden round filter paper discs (12 mm diameter) into one of the holes. To ensure constant total flow and humidity (65% r.h., 24°C) prior and during stimulation the alternating second flow channel of a Syntech CS-05 Stimulus Controller was connected via identical tubing and pipettes to the other hole. The humidity was measured at the tube outlet prior recordings, using a digital thermo-hygrometer (P330, Tematec GmbH, Hennef, Germany).

Compounds to be tested were applied to the filter paper discs in 10µl solvent, which was allowed to evaporate for 1min prior to stimulation. CO_2_ was applied by filling a Pasteur-pipette (2.5mL) with 20% CO_2_, through which 4mL air were pushed during stimulation and mixed with 8mL air from the constant flow, resulting in a final concentration of approximately 4%. When water was used as solvent or stimulus, humidity increased to 66% r.h. at 24°C during stimulation. Prior to stimulation and after each 10th puff, the vigor of the preparation was tested. Breath was used as positive control, as contained humidity and CO_2_ elicited reliable responses. The average lifetime of the preparations exceeded 10hrs, but preparations were discarded earlier if the response to breath fell below 80% of the initial response, or after all compounds had been tested three times. All stimuli (see below) were applied in randomized order. In total, every compound was tested 15 times on 6 animals (1–3 replicates per animal). For statistical analysis and graphical display responses to the respective solvent were subtracted from responses to the stimuli.

Statistical analysis and graphical charts were implemented using the statistic program “R” (R version 2.9.2 [38] (2009-08-24)). Square-root transformed data showed optimally reduced variance heterogeneity among treatments and were successfully tested for normality (“R” command “qqnorm”). Transformed data of EAG/EPG responses were compared separately for each head appendage to responses to the respective solvent, applying Welch two sample t-tests.

### Test compounds and solvents

Stimulants are selected by their known ecological function in soil-inhabiting insects or occurrence in plant root exudates, and by their structure and carbon chain length in order to test a broad range of chemically diverse compounds. Exponents given for each chemical indicate the purchasing source mentioned below.

1. Compounds attractive or repellent to other soil-dwelling insects. Gases: CO_2_, terpenoids: (+)-camphene^1)^, (−)-camphene^2)^, β-elemene^3)^, α- and β-farnesene (mix of isomeres)^1)^, (−)-limonene^1)^, (+)-limonene^2)^, linalool (mix of enantiomers)^1)^, β-myrcene^2)^, α-pinene^2)^, β-pinene^1)^, α-terpinene^2)^, α-phellandrene^1)^; others: benzaldehyde^1)^, ethanol^4)^, ethyl acetate^1)^, hexyl acetate^1)^ [39];
2. Compounds commonly released by plant roots. Acids: acetic acid^1)^, citric acid^1)^, formic acid^2)^, fumaric acid^5)^, lactic acid^2)^, malic acid^4)^, oxalic acid^1)^, propionic acid^1)^ [28], [30]; terpenoids: β-caryophyllene^2)^, eucalyptol (1,8-cineol)^2)^, γ-terpinene^1)^ [40].
3. Other compounds: acetone^1)^, 2-butanone^1)^, butyl acetate^2)^, butylamine^2)^, α-(−)-cedrene^2)^, cinnamaldehyde (cinnamal)^1)^, hexylamine^2)^, hydrochloric acid^4)^, ethanal^1)^, methanal^4)^, methanol^4)^, methyl acetate^2)^, 1-nonanol^2)^, 1-octanol^1)^, pentylamine^2)^, propanal^1)^, 1-propanol^4)^, propyl acetate^2)^, propylamine^1)^, pyridine^6)^, sulcatone^1)^.

Acids were dissolved in dichloromethane (DCM) supplemented by 20% water to increase solubility (the applied concentration was 1µg/µl). Remaining compounds other than CO_2_ were diluted in DCM^4)^ and used at 1µg/µl. DCM supplemented by 20% water (for acids), clean filter paper (for undiluted compounds and CO_2_) and DCM (for remaining compounds) served as controls, respectively.

Components were purchased from ^1)^ Sigma Aldrich (Steinheim, Germany), ^2)^ Fluka (Steinheim, Germany), ^3)^ Aapin Chemicals Limites (Abingdon, Oxfordshire, UK), ^4)^ Roth (Karlsruhe, Germany), ^5)^ Alfa Aesar (Karlsruhe, Germany) and ^6)^ Merck (Darmstadt, Germany).

## Results

### Scanning and transmission electron microscopy (SEM & TEM)

**Figure 1:**
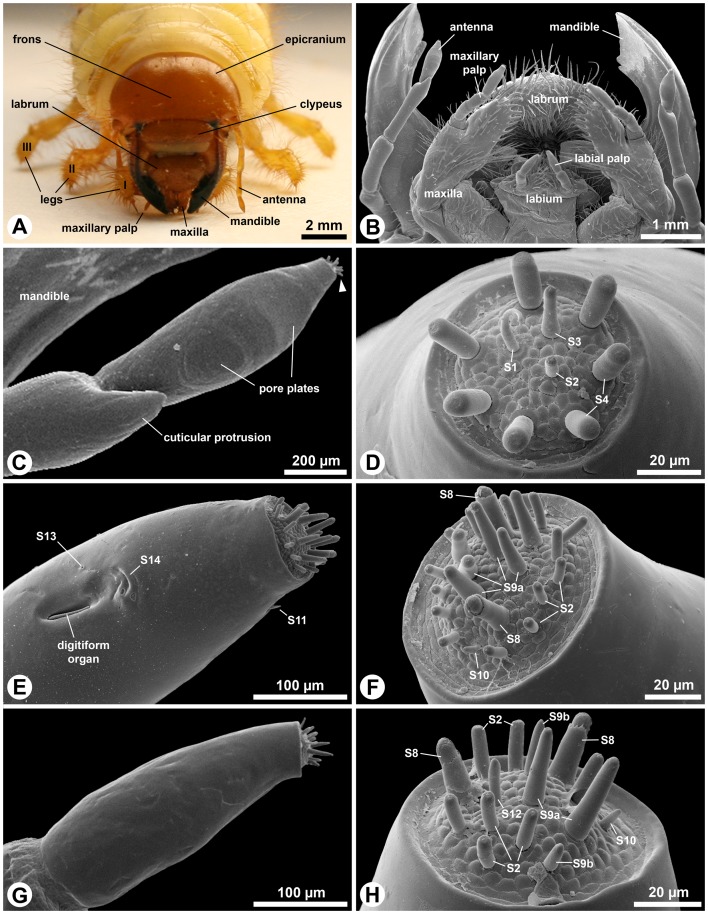
Gross morphology of head and mouthpart appendages of third instar *Melolontha melolontha* larvae. A: Macro photograph. Frontal view on the head and the anterior body. B–H: SEM. B: Ventral view on the larval mouthparts showing labium and maxillae with their palps. In this specimen, the antennae are held below the opened mandibles, thus they become visible in this viewing angle. C: The apical segments of the antenna. The subapical segment bears a conical cuticular protrusion on its antero-lateral margin. Note the small apical sensilla field (arrowhead). Pore plates are hardly visible. D: Frontal view on the apical sensilla field of the antenna. This specimen possesses seven S4 sensilla. E: Tip of the apical segment of the maxillary palp. On this appendage, several different sensilla occur also below the apical sensilla field. F: The apical sensilla field of the maxillary palps bears the highest number of sensilla among the head sensory organs. G: The apical segment of the small labial palps. H: The apical sensilla field of the labial palps.

The antennae of third instar *M. melolontha* consist of five, and the maxillary and labial palps consist of four and three segments, respectively (length ratio antenna: maxillary palp: labial palp  = 20∶7∶4) (Fig. 1B). While all appendages possess conspicuous crown-like apical sensillum fields (Figs. 1C–H), only antennae and maxillary palps carry additional subapical sensilla, namely three pore plates on the sides of the apical antennal segment (Figs. 1C, E), small peg-like sensilla and one pore plate on a cuticular protrusion of the post-apical antennal segment and the digitiform organ on maxillary palps. In total, 17 different sensory organs are present on larval head appendages (see Table 1).

**Table 1:** Hypothesized function, abundance external morphology and dendritic structure (ODS: outer dendritic segment, TB: tubular body) of sensilla on antennae (A) and maxillary (M) and labial palps (L) in *Melolontha melolontha* larvae.

Hypothesized function	Sensillum (type, figure)	Number (location)	Structure	Surface	Pores	Dendrites	Reference
	Pore plates (placoid, Figs. 1,3,4)	4 A (3 lateral on apical segment, 1 on subapical segment)	large, shallow cuticular depression	smooth	perforated with minute and larger pores	approx. 300 bundles of 3–4 branching ODS	CO_2_ [5]; olfaction [11], [17], [18], this study
Olfaction	S7 (basiconic, Figs. 3,7)	1 A (subapical segment)	small, distally tapering seta	slightly sculptured	wall pores	2–3 ODS (branches?)	this study
	S10 (basiconic, Figs. 1,9)	1 M (apex), 1 L (apex)	small, conical seta	slightly sculptured	wall pores	2 branching ODS	this study
	S2 (chaetic, Figs. 1,5)	1 A (apex), 14 M (apex), 7 L (apex)	small, slightly conical seta with blunt tip	smooth to slightly sculptured	terminal pore	4–5 ODS (one with TB)	this study
	S3 (chaetic, Figs. 1,6)	1 A (apex)	large, slender, conical seta with blunt tip	smooth	terminal pore	4–5 ODS	this study
Contact chemo-reception or bi- modal (tubular body indicates additional mechanoreception)	S4 (chaetic, Figs. 1,6)	7–8 A (apex)	large, thick, cylindrical seta with blunt tip	slightly sculptured	terminal pore	5–6 ODS (one with TB)	this study
	S5 (chaetic, Figs. 3,7)	5 A (subapical segment)	small, egg-shaped seta in large circular socket	smooth	terminal pore	unknown	this study
	S8 (chaetic, Figs. 1,8)	2 M (apex), 2 L (apex)	large, thick, conical seta with spherical apex and collar	slightly sculptured	terminal pore	4–5 ODS (TB not observed)	this study
	S9a (chaetic, Figs. 1,8)	5–6 M (apex), 2 L (apex)	large, slender, conical seta with blunt tip	smooth	terminal pore and lateral molting pore	7 ODS (one likely with TB)	this study
	S9b (chaetic, Figs. 1,8)	2 L (apex)	small, slender, conical seta with blunt tip	smooth	terminal pore	7 ODS (one likely with TB)	this study
Hygro-/thermo-reception	Digitiform organ (coeloconic, Fig. 2)	1 M (lateral on apical segment)	sunken seta in longish cuticular recess	smooth	aporous	1 ODS (distally lamellate)	CO_2_ [14]; mechano-reception [21], [22], [24]; hygro-/thermoreception [13], [21], this study
	S12 (chaetic, Figs. 1,10)	1 L (apex)	small, slender, cylindrical seta with blunt tip	smooth	subterminal pore	1 ODS (distally lamellate)	this study
	S1 (trichoid, Figs.1,5)	1 A (apex)	thin, bent seta with bifurcated tip	slightly grooved	basal molting pore	2 ODS (TB not observed)	this study
Mechanoreception	S11 (trichoid, Figs. 1,10)	1 M (apex)	small, distally tapering seta	smooth	aporous	1 ODS with TB	this study
	S13 (not classi-fiable, Fig. 2)	1 M (lateral on apical segment)	small pit	smooth	aporous	1 ODS with TB	this study
	S14 (not classi-fiable, Fig. 2)	3–4 M (lateral on apical segment)	flat, bent cuticular furrows	smooth	unknown	unknown	this study
Unknown	S6 (chaetic, Figs. 3,7)	1 A (subapical segment)	small, blunt seta	smooth	unknown	unknown	this study

### Digitiform organ and adjacent sensilla (S13 and S14)

**Figure 2:**
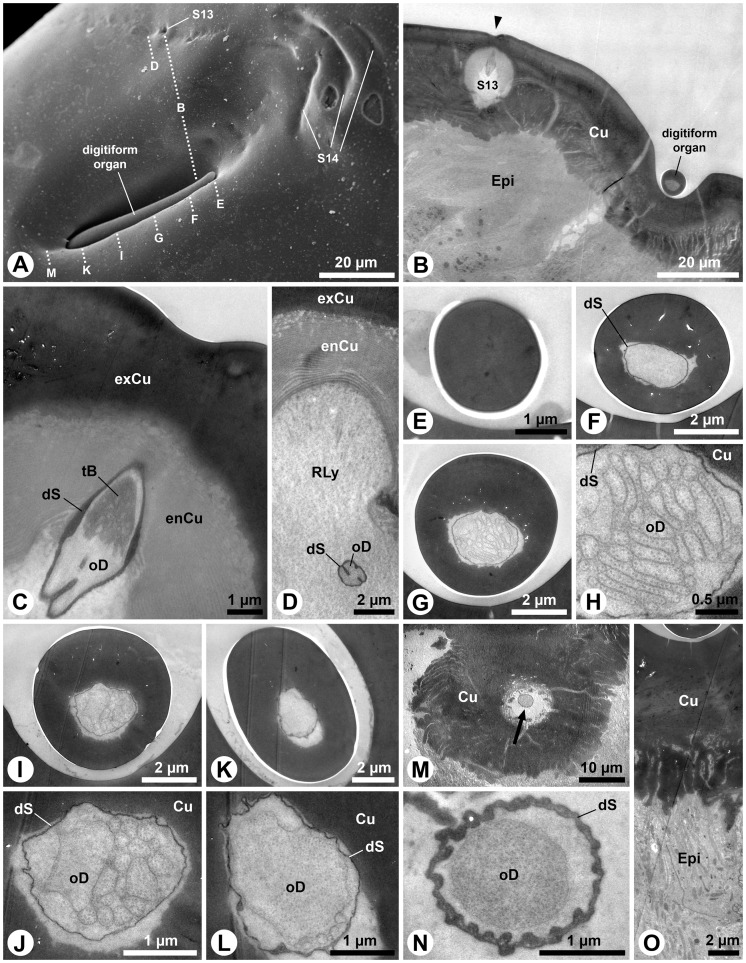
Digitiform organ and adjacent sensilla on *M. melolontha* larval apical segment of the maxillary palps. A: SEM. The digitiform organ is situated on the bottom of a cuticular depression. Note a row of flat pits (S13) and bent furrows (S14). Dotted lines indicate approximate cutting planes of transverse sections shown in figures B, D–G, I, K and M. B–O: TEM. B: Section on the level of the anterior third of the digitiform organ. In addition to the digitiform organ, one S13 is cut obliquely (arrowhead: flat cuticular pit above S13). C: Magnification of S13. An ensheathed tubular body is embedded in the matrix of the endocuticle. D: A further posterior section shows the single ensheathed outer dendritic segment of the S13 sensilla projecting through its receptor lymph cavity. E: Transverse section of the massive aporous tip of the digitiform organ. F: Posterior of the tip, the shaft lumen houses a thin dendritic sheath, which is empty at this section level. G: Outer dendritic segments occur within the middle portion of the digitiform organ. H: Note the lamellar arrangement of the flattened outer dendritic segments. I: Further posteriorly, the number of outer dendritic segments is reduced. J: The profiles of the outer dendritic segments are either round or enlarged polygons. K, L: Close to the base only few outer dendritic segments are observable, M: The socket of the digitiform organ is formed by sclerotized cuticle. Note the outer dendritc segment in the central lumen (arrow). N: Only one outer dendritic segment is present, surrounded by a thick and slightly folded dendritic sheath. O: The integument below the digitiform organ. Abbr.: Cu, cuticle; dS, dendritic sheath; Epi, epidermis; enCu, endocuticle; exCu, exocuticle; oD, outer dendritic segment; RLy, receptor lymph; S13–14, sensilla 1–14; tB, tubular body.

The digitiform organ, which is presumably a hygro-thermoreceptor (cp. Table 1), is located on the lateral surface of the apical segment of the larval maxillary palps (Fig. 1E). It consists of a long, distally slightly tapering seta, which lays flat in a longish oval recess of the palpal cuticle (Fig. 2A, B). Its blunt tip points towards the apex of the maxillary palp, and it consists of a massive, poreless cuticle (tip: Fig. 2E) with longitudinal channels (shaft: Figs. 2F, G). Subapically, the shaft lumen contains a thin dendritic sheath without dendritic structures (Fig. 2F). However, numerous flat dendritic profiles, partly arranged in a lamellar way, reside inside the dendritic sheath in the center of the organ (Figs. 2G, H). Their number is reduced towards the base of the shaft, but several profiles gain in diameter (Figs. 2 I-L). Finally, only one ensheathed outer dendritic segment is present in the socket (Figs. 2 M, N). All profiles in the shaft are branches of this single dendrite. The socket does not show flexible cuticle areas (Fig. 2M). The integument of the recess does not show any structures, indicative of additional sensory functions (Fig. 2O).

Adjacent to the digitiform organ on the maxillary palps two further sensillum types are identified: the S13 and S14 sensillum (Fig. 1E; 2A). The S13 sensillum is characterized by a small, flat cuticular depression (Fig. 2B). A single, ensheathed outer dendritic segment, terminating in a large tubular body is projecting through a cuticular channel towards the cuticular depression (Figs. 2B–D). The dendritic sheath terminates in the matrix of the endocuticle (Fig. 2C). The putative S14 sensilla represent a group of bent cuticular furrows above the digitiform organ (Figs. 1E; 2A). Their ultrastructure is not known.

### Pore plates

**Figure 3:**
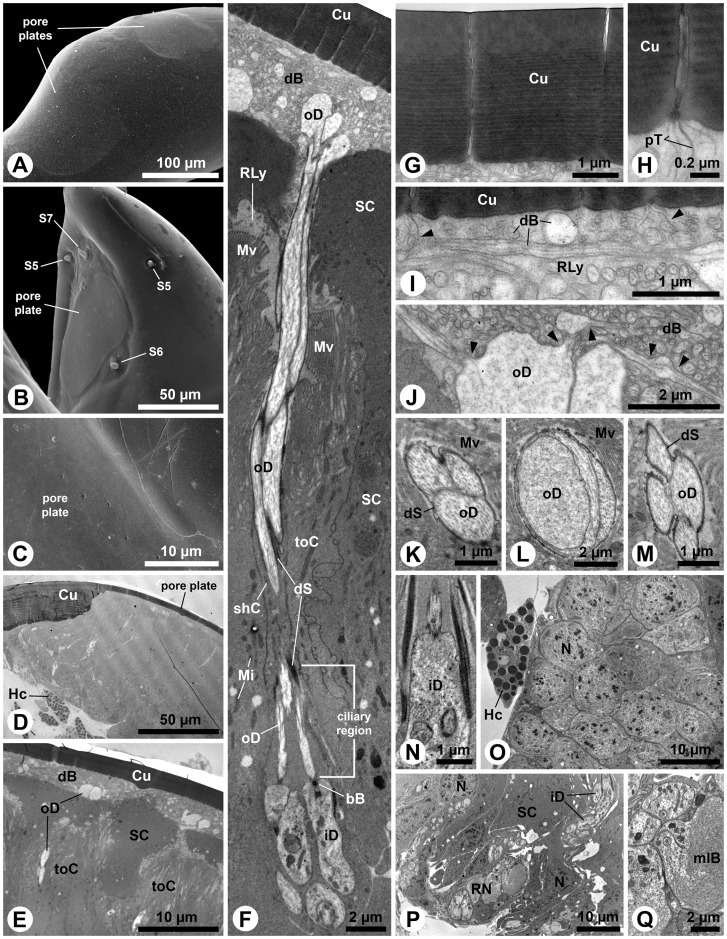
Antennal pore plates of third instar *M. melolontha* larvae. A–C: SEM. A: Two pore plates on the apical segment. B: Pore plate and adjacent sensilla (S5–7) in the lateral protrusion of the subapical segment. C: Pore plate and adjacent cuticle intersection. Apart from occasional openings (see Fig. 4), the surface of the pore plate appears smooth. D–Q: TEM. D: Panoramic view of a transverse section, displaying the thin pore plate cuticle and the large tissue cluster below. E: Layered arrangement of different cell types below the pore plate cuticle. F: Three outer dendritic segments, originating from the inner dendritic segments, deflect towards the pore plate. Note the relatively short ciliary portion of the outer dendritic segments. G: The pore plate cuticle, penetrated by narrow channels. H: Internally, each channel exhibits a bundle of tubules. I: The tubules contact small dendritic branches (arrowheads). Note the horizontal dendritic branch, originating from a larger profile (bottom right). J: Dendritic profiles with different diameters and branching points (arrowheads) below the pore plate cuticle. K–M: Transverse sections of outer dendritic segment bundles, showing profiles of varying number, diameter and shape. N: Formation of the dendritic sheath around the apex of an inner dendritic segment. O: Cluster of receptor neuron somata close to the central hemolymph space of the apical antennal segment containing a hemocyte. P: Supporting cells surround somata and inner dendritic segments. Q: Region of the receptor somata from where inner dendritic segments protrude with large multilamellar body. Abbr.: bB, basal body; Cu, cuticle; dB, dendritic branch; dS, dendritic sheath; HC, hemocyte; iD; inner dendritic segment; Mi, mitochondrion; mlB, multilamellar body; Mv, microvilli; N, nucleus; oD, outer dendritic segment; pT, pore tubule; RLy, receptor lymph; RN, receptor neuron; S13–14, sensilla 13–14; shC, sheath producing cell; SC, support cell; toC, tormogen cell.

Four olfactory pore plates are present on the antennae of third instar *M. melolontha* larvae. Three with average diameters of about 100–200µm are located on the ventral and dorsal surfaces of the apical segment (Figs. 1C; 3A) and one of about (25µm in width and 70µm in length) is located on the inner surface of the lateral protrusion of the subapical segment (Fig. 3B). Sections show that the cuticle of a pore plate is almost six times thinner than adjacent parts of the antennal cuticle (Fig. 3D). A large tissue cluster of distinct cell types is present below each pore plate (Fig. 3E). Among them are numerous sensorial units, each consisting of a bundle of ensheathed dendrites, projecting radially towards the thin pore plate cuticle (Fig. 3F). These more or less columnar sensory units are surrounded and separated by support cells (Figs. 3E, F). The average distance between adjacent dendrite bundles is about 15µm.

Over all, the sensory units exhibit a clear stratified arrangement (Figs. 3E, F–Q). Numerous fine pores penetrate the pore plate cuticle (Figs. 3F, G). Contrary to the name of this structure, surface openings appear to be sparse (Fig. 3C). However, dozens of fine pores are detectable in each ultrathin section (Fig. 3F). Electron-dense tubules are associated with the pores (Fig. 3G). These tubules extend into the space below the cuticle (Fig. 3H), where they get in close vicinity to hundreds of fine dendritic branches with diameters between 0.1–0.3 µm (Fig. 3I). They form a flat, lenticular receptor area directly below a fraction of the pore plate (Fig. 3F, I). These fine branches originate from medium sized dendritic branches with diameters between 0.5–1 µm (Figs. 3I, J). The latter branch off from the inflated apices of three-to-four outer dendritic segments (Fig. 3F, J–M). A thin dendritic sheath surrounds the outer dendritic segments, which do not have ciliary character (Figs. 3F, K–M). The sheath is formed in the region where the outer dendritic segments project as short cilia out of the inner dendritic segments (Fig. 3F, N). The inner dendritic segments originate from clusters of sensory cell bodies that are located close to the central hemolymphatic space of the antennae at the base of the tissue cluster below the pore plate (Figs. 3O–Q).

**Figure 4:**
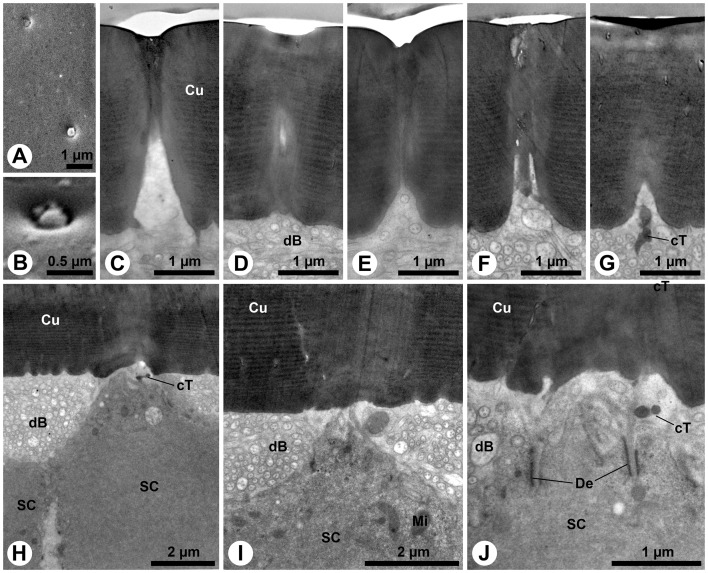
Structure of the pore-like openings and support cells of the antennal pore plates of *M. melolontha* larvae. A, B: SEM. A: Here the pore-like openings are plugged. Note small dark spots spread over the surface. B: Higher magnification of a plug within a pore-like opening. C–J: TEM. C: Longitudinal section of a pore-like opening. Although the pore-plate cuticle is fully ruptured by the hour-glass-like duct, its outer half seems to be sealed. D: In this oblique section the duct appears somewhat oval. E: Dendritic branches project into the inner half of the duct. F: This section shows a cuticular protrusion in the duct. G: This protrusion extends as a cuticular thread between the dendritic branches. H: The epidermal support cells have punctual contacts with the pore-plate cuticle. This separates adjacent areas with dendritic branches. I: Mitochondria and electron-dense material are concentrated in the contact areas of the support cells. J: Desmosome-like densities can be observed in the apical membranes of the support cells. Abbr.: cT, cuticular thread; Cu, cuticle; dB, dendritic branch; De, desmosome; Mi, mitochondrion; SC, support cell.

The aforementioned wider openings (Fig. 3C) are often plugged or sealed (Fig. 4A, B). The pore plate cuticle is penetrated by hour-glass-like ducts, in which the sealing material can often be seen in the outer part (Fig. 4C). The ducts are relatively narrow in the middle of the cuticle (Fig. 4D). Outer dendritic segments project into the inner openings of the ducts (Figs. 4E–F). Often cuticular threads protrude from the duct lumen between the outer dendritic segments (Figs. 4F, G). Close to these ducts, punctual contacts between support cells and the pore plate cuticle occur (Fig. 4H). Electron-dense material and mitochondria are concentrated in such contact areas (Fig. 4I) and desmosome-like densities are visible at the apical membrane (Fig. 4J).

### Peg-like sensilla on apical fields and in antennal protrusion

**Figure 5:**
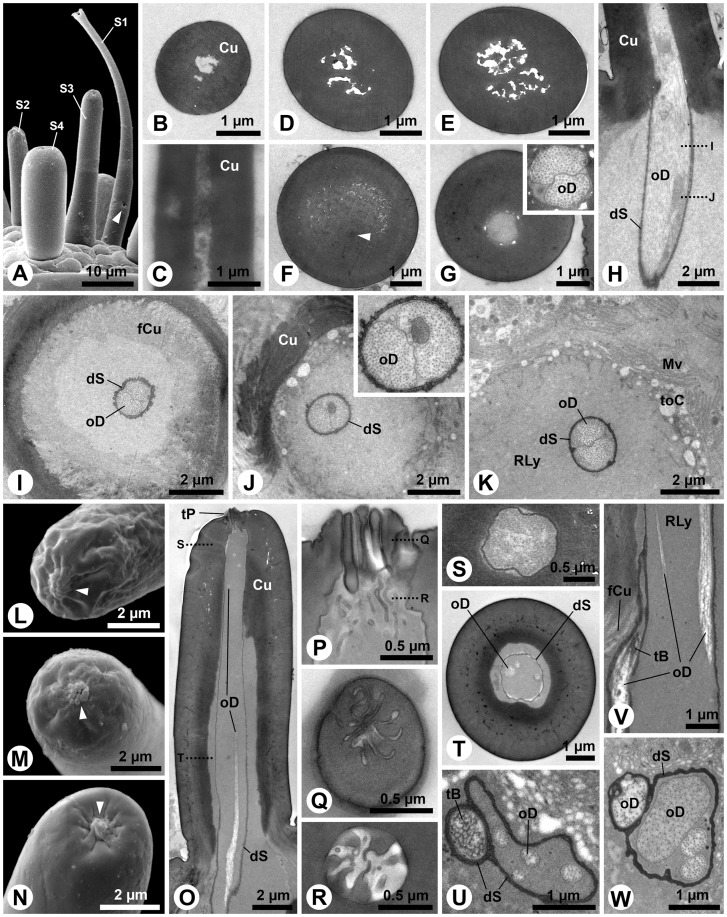
S1 and S2 sensilla of *M. melolontha* larval antennae. A: SEM. Four different setiform sensilla on the apical sensilla field with putative molting-pore (arrowhead) on S1. B–K: TEM. B: Transverse sections of apical S1. The empty lumen is irregularly shaped. C: Transverse section of S1 center with spongiously hollow shaft. D, E: Further basally, the spongious area enlarges. F: Closely above the socket, the cuticle expands, reducing spongious areas. Note the electron dense spot (arrowhead). G: Closely above the socket, two outer dendritic segments are present (inset: 2× magnification of dendrites). H: This oblique longitudinal section shows the innervation of the sensillum base (dotted lines: approximate cutting planes for Figure I, J).I: Transverse section of the S1 socket revealing its flexible cuticle. J: An electron-dense structure, most likely a vesicle filled with granular material (compare with [76]) is present in one dendrite (inset: 2.5× magnification). K: Transverse section below the socket. L–N: SEM. L–N: Tips of S2 on antenna, maxillary palp and labial palp with finger-like protrusions (arrowheads). O–W: TEM. O, P: Longitudinal section of labial palp S2 (dotted lines: approximate cutting planes for Figures S, T) and magnification of the pore region (dotted lines: approximate cutting planes for Figures Q, R). Q: Transverse section of S2 apex. R: Transverse section below the pore demonstrating lumen bound cuticular threads. S: An empty dendritic sheath is present in the lumen. T: Outer dendritic segments at the base of the shaft. U: Five outer dendritic segments in a S2 socket, one containing a tubular body. V: Longitudinal section depicting the attachment of the tubular body to the socket cuticle. W: Four outer dendritic segments are present in this S2. Abbr.: Cu, cuticle; dS, dendritic sheath; fCu, flexible cuticle; Mv, microvilli; oD, outer dendritic segment; RLy, receptor lymph; S1–4, sensilla 1–4; tB, tubular body; toC, tormogen cell; tP, terminal pore.

The S1 sensillum is the longest sensillum of the antennae and occurs in the centre of the apical antennal sensilla field (Fig. 5A). The single, slightly bent seta has a bifurcated tip (Figs. 1D; 5A). A spongiform lumen is observed in the distal two thirds of its slender, poreless shaft (Figs. 5B–E). The cuticle becomes denser in the basal third (Fig. 5F). Shortly above the socket, two ensheathed outer dendritic segments occur inside the narrow lumen (Figs. 5G). Following the innervation deeper does not reveal numeric changes in the dendritic pattern (Figs. 5H–K). The socket itself bears areas with flexible cuticle (Figs. 5I, J). A tormogen cell with a well-developed apical microvilli border surrounds the dendrite below the socket (Fig. 5K).

The S2 sensillum, which is the only sensillum type in common of all three head appendages (Figs. 1D, F, H), is relatively small. It occurs once in the centre of the apical sensillum field of the antennae (Figs. 1D; 5A), 14 times in the periphery of the apical sensillum field of the maxillary (Fig. 1F) and 7 times in the periphery of the apical sensillum field of the labial palps (Fig. 1H). Preparation artifacts may account for minor variations of tips and surfaces among appendages (Figs. 5L–N). However, all sensilla classified as S2 are of similar size and have a single terminal pore (Figs. 5L–N) and a poreless shaft (Figs. 5O, T) in common. The terminal pore is formed by densely arranged finger-like cuticular protrusions (Fig. 5P). Slit-like interspaces between the protrusions (Fig. 5Q) merge in the central lumen of the sensillum (Fig. 5R). Thin cuticular threads project from the protrusions into the lumen (Figs. 5P, R). A subapical transverse section reveals a thin dendritic sheath without dendritic segments inside the narrow lumen (Figs. 5O, S). Further basally, the lumen becomes wider and the dendritic sheath houses dendritic segments (Figs. 5O, T). Four-to-five outer dendritic segments innervate the S2 sensillum (Figs. 5U–W). One of them always terminates as a tubular body (Figs. 5U, V), attached to flexible cuticle areas of the socket (Fig. 5V). An individual dendritic sheath always separates the single tubular body-forming dendrite from the other ones (Figs. 5U–W), which proceed into the shaft (Fig. 5O, V).

**Figure 6:**
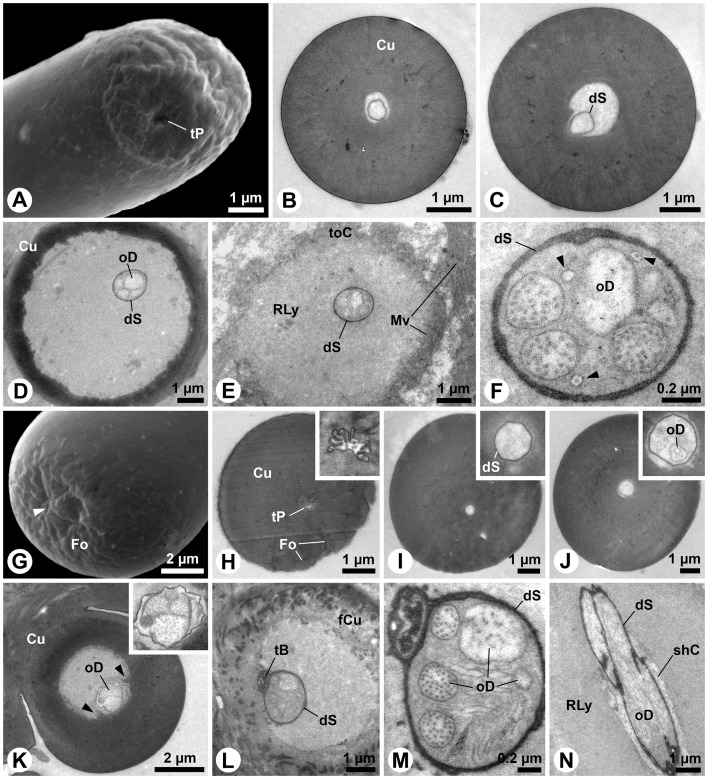
S3 and S4 sensilla on *M. melolontha* larval antennae. A: SEM. Tip of the S3 sensillum. B–F: TEM. B: Transverse sections of the apical part of S3. The central lumen contains an empty dendritic sheath. C: Transverse section of the middle part of S3. The empty dendritic sheath follows a furrow along the inner surface of the shaft. D: Transverse section of the socket of S3 revealing several ensheathed outer dendritic segments. E: Below the socket, the dendrites are surrounded by the receptor lymph producing tormogen cell. F: Some dendritic profiles show microtubules. Note the small profiles (arrowheads). G: SEM. Tip of a S4 sensillum with a terminal pore (arrowhead). H: Oblique section of the pore area of S4 (inset: 3.5× magnification of the pore). I: Subterminal transverse section of the same sensillum. The narrow lumen (4× magnification see inset) contains a thin dendritic sheath but no observable dendrites. J: Transverse section of the center of the sensillum shaft. The still narrow lumen (inset: 3× magnification) houses at least one outer dendritic segment. K: Oblique transverse section of the area where the socket (top left) extends into the shaft (lower right). The dendritic sheath contacts the shaft cuticle (inset: 2× magnification of dendrites). Two cuticular lamellae (arrowheads) flank the dendritic sheath. L, M: Transverse section of the socket, containing a dendritic sheath attached to the cuticle. One dendrite terminates in a tubular body. Not all dendrites exhibit clear microtubules. N: Oblique longitudinal section of the innervation of a S4 sensillum. The dendritic sheath is adjacent to the extensions of its origin, the thecogen cell. Abbr.: Cu, cuticle; dS, dendritic sheath; fCu, flexible cuticle; Fo, fold; Mv, microvilli; oD, outer dendritic segment; RLy, receptor lymph; shC, sheath producing cell; tB, tubular body; toC, tormogen cell; tP, terminal pore.

The S3 sensillum is relatively large and exclusively located in the centre of the antennal apex (Figs. 1D; 5A). Its blunt tip bears a laterally shifted subterminal pore (Fig. 6A). The poreless shaft consists of thick cuticle (Figs. 6B, C). Apically, the narrow lumen houses a dendritic sheath (Fig. 6B). Further basally, the lumen is wider and the dendritic sheath follows a lateral fold in the shaft cuticle (Fig. 6C). Four-to-five outer dendritic segments innervate this sensillum (Figs. 6D–F). Some dendritic segments show numerous microtubules. Interestingly, very small profiles containing microtubules can be observed as well (Fig. 6F).

The thick, cylindrical S4 sensillum also occurs exclusively on the antenna and constitutes the peripheral ring of the apical sensilla field (Fig. 1D). Pore structures are hardly visible (Fig. 6G) but a small terminal pore becomes visible in sections (Fig. 6H). Similar to the S2 sensillum, the S4 terminal pore possesses small finger-like protrusions and thin cuticular threads (inset in Fig. 6H). Furthermore, the subapical dendritic sheath and outer dendritic segments are present in the narrow lumen of the massive, poreless shaft (Figs. 6I, J). Close above the socket, the dendritic sheath is paralleled by two cuticular lamellae (Fig. 6K). Four-to-five outer dendritic segments extend into the shaft lumen (inset in Fig. 6K). Inside the socket, the dendritic sheath is attached to flexible cuticle parts (Fig. 6L). A dense tubular body is formed by one separated dendrite (Figs. 6L, M). Protrusions of the sheath producing thecogen cell can be observed below the socket (Fig. 6N).

**Figure 7:**
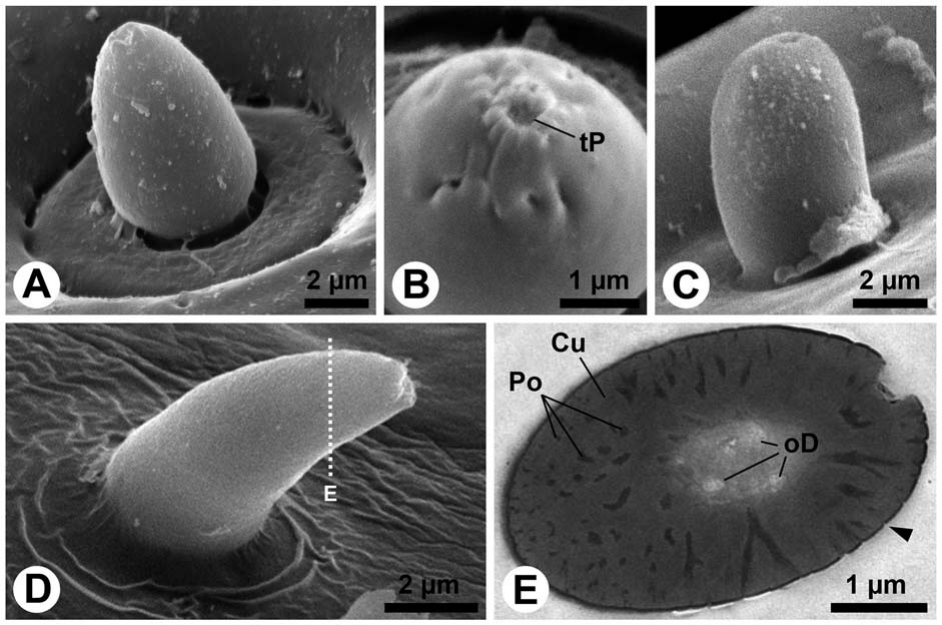
S5, S6 and S7 sensilla of the antennae of *M. melolontha* larvae. A–D: SEM. A: Lateral view on the egg-shaped S5 sensillum. Note the large, circular socket. B: Higher magnification of the tip of a S5 sensillum. Finger-like cuticular projections surround a terminal pore. C: Lateral view on a short, blunt S6 sensillum. D: S7 sensillum with a short, conical, bent shaft. Its tip seems to be damaged. The dotted line indicates the approximate cutting plane of the transverse sections shown in Figure E. E: Oblique transverse section of the S7 sensillum. The cuticle of the sensillum is penetrated by numerous pores which connect the outside with the lumen, where outer dendritic segments are present. Note the minute pore openings (arrowhead). Abbr.: Cu, cuticle; oD, outer dendritic segment; Po, pore; tP, terminal pore.

Sensillum types S5, S6 and S7 are located inside the lateral protrusion of the subapical antennal segment, close to the pore plate (Fig. 3B). S5 is a small, egg-shaped sensillum in a comparatively large circular socket (Fig. 7A). It possesses a terminal pore surrounded by fine finger-like protrusions, similar to those of the S2 sensillum. The S6 sensillum is also very small, but its socket is inconspicuous (Fig. 7C). The ultrastructure of S5 and S6 is not yet known. The S7 sensillum is a short, slightly bent, conical seta with a slightly sculptured surface (Fig. 7D). Sections reveal the porous shaft structure of this sensillum (Fig. 6E). At least three outer dendritic segments could be observed inside the shaft lumen (Fig. 6E).

**Figure 8:**
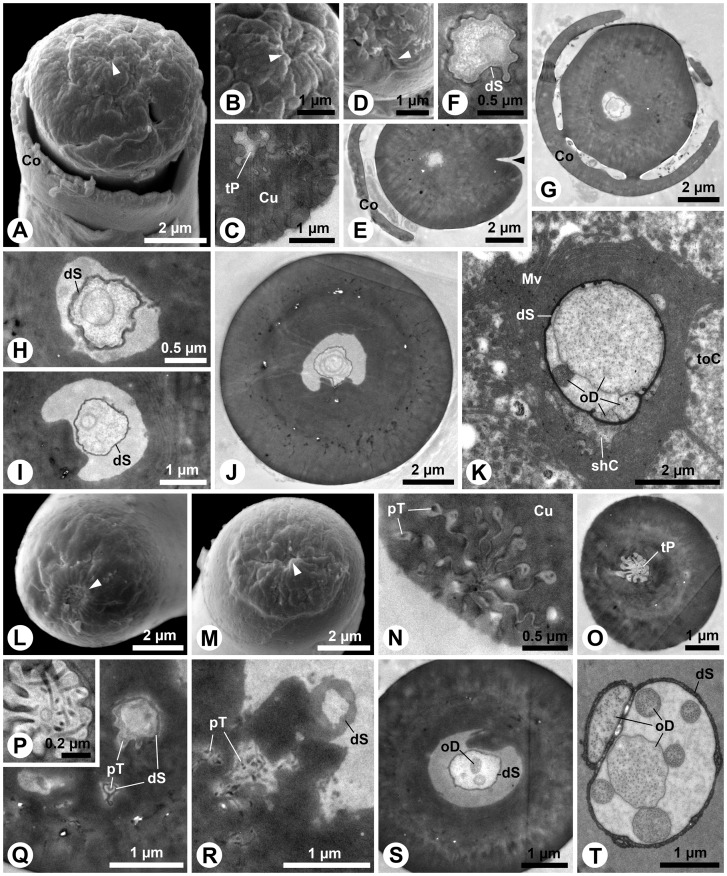
S8 and S9 sensilla on *M. melolontha* larval palps. A–B: SEM. A: Tip of a S8 sensillum from the maxillary palp with inconspicuous terminal pore (arrowhead) and conspicuous opening (see Figures D, E). B: Magnification of the terminal pore (arrowhead), surrounded by cuticular protrusions. C: TEM. Oblique section of the terminal pore area. D: SEM. The conspicuous opening (arrowhead) is just a deep fold. E, F: Oblique transverse section of the S8 sensillum on the level of the fold (arrowhead). Parts of the collar are visible on the left. A dendritic sheath but no dendritic elements are observable. G, H: Further posterior section of the collar origin, revealing membranous structures in the lumen. I: The dendritic sheath in the shaft center extends along a cuticular lamella, generating a crescent lumen. J: The dendritic sheath in the S8 shaft is very closely allied to the lamella. K: Transverse section below the socket. In this specimen the dendritic sheath encloses four outer dendritic segments. One of them contains conspicuously dense arranged microtubules. L, M: SEM. L: Tip of the S9 sensillum of the maxillary palp. Finger-like protrusions surround the pore (arrowhead). M: S9 with an elevated terminal pore (arrowhead) region. N–T: TEM. N: Oblique section of the pore region with putative pore tubules adjacent to the protrusions. O, P: Lumen below the terminal pore. Magnification reveals streaks of electron-dense material. Q: Channels with a thin lining (putative dendritic sheath) below the tip of S9. R: Putative pore tubules extend towards the central lumen. A dendritic sheath is attached to a cuticular lamella. S: Transverse section of a S9 base with ensheathed outer dendritic segments. T: Seven outer dendritic segments are present below the socket. Abbr.: Co, collar; Cu, cuticle; dS, dendritic sheath; Mv, microvilli; oD, outer dendritic segment; pT, pore tubules; shC, sheath producing cell; toC, tormogen cell; tP, terminal pore.

S8 is the largest sensillum type on maxillary and labial palps. It occurs twice in the central area of the apical sensillum fields of both appendages (Figs. 1F, H). A peculiar tip, formed by a nearly spherical apex, which is surrounded by a cuticular collar, characterizes this sensillum (Fig. 8A). Besides a relatively inconspicuous terminal pore surrounded by finger-like protrusions (Figs. 8A–C), these sensilla show conspicuous cuticular openings (Fig. 8A), which turn out to be only deep cuticular folds (Fig. 8D, E). The terminal pore merges into the central lumen of the shaft where a dendritic sheath is present (Figs. 8E, F). Subapically, membranous structures are present inside the sheath (Figs. 8G–J). The thick shaft cuticle is poreless (Figs. 6G, J). Longitudinal channels are present in the cuticle (Fig. 8J). Basally, the sheath is guided by a cuticular lamella (Figs. 8I, J). Four-to-five outer dendritic segments innervate the S8 sensillum (Fig. 8K). Although one of them contains densely arranged microtubules, clear evidence for the presence of a mechanosensory tubular body is lacking.

The second largest sensillum on both palps belongs to type S9. Although structurally very similar among the appendages, two morphological variations of this type could be identified: the large S9a and smaller S9b. Five-to-six S9a occur on the maxillary palp (Fig. 1F) and two on the labial palp (Fig. 1H). The smaller S9b occurs twice on the labial palp (Fig. 1H). All S9 possess terminal pores, often inconspicuous (Fig. 8L), but sometimes a little elevated (Fig. 8M). The terminal pores bear finger-like protrusions, but unlike in the previously described sensilla, interspaces between these protrusions contain electron-dense tubules (Fig. 8N). The tubules from the terminal pore extend into the central lumen (Figs. 8 O, P). A peculiar feature of these sensilla is the presence of additional channels with tubules that originate laterally of the terminal pore and project radially from the tip towards the central lumen of the shaft (Figs. 8Q, R). A dendritic sheath is attached to a cuticular lamella in the lumen (Fig. 8R). Outer dendritic segments are present in the basal part of the sensillum (Fig. 8S). Up to seven dendrites, one in a separate sheath innervate S9 sensilla (Fig. 8T). Comparing this with the findings for sensilla S2 (see Figs. 5U, W) and S4 (see Fig. 6M) indicates that the separated dendrite may contain a tubular body in its tip.

**Figure 9:**
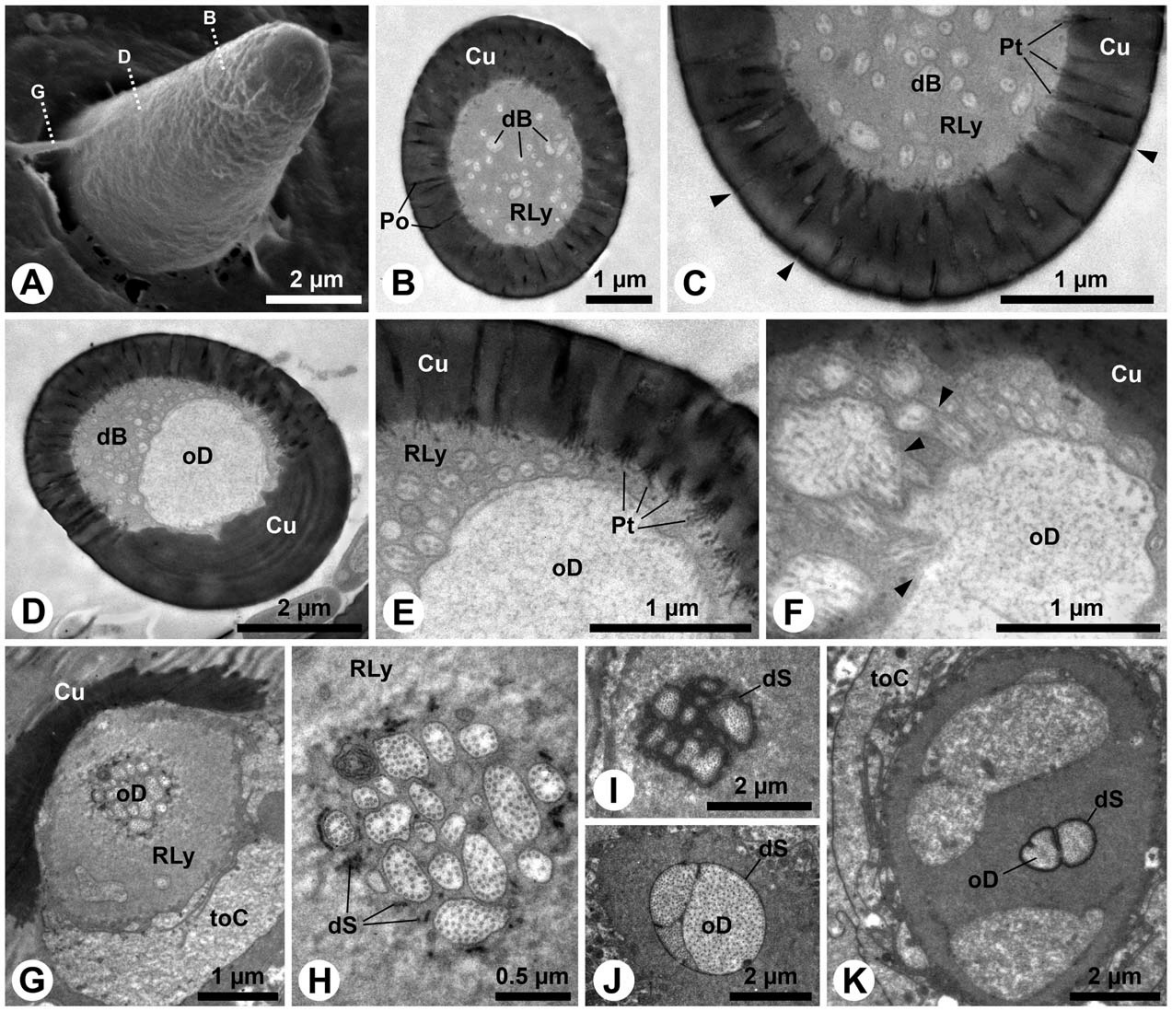
S10 sensillum of the palps of *M. melolontha* larvae. A: SEM. S10 sensillum from the maxillary palp. The surface is slightly sculptured. Dotted lines indicate approximate cutting planes of transverse sections shown in Figures B, D and G. B–K: TEM. B: Oblique transverse section of the apical part of the shaft. The cuticle is porous and the wide lumen is sparsely filled with thin dendritic branches. C: Bundles of short pore tubules are directed towards the lumen of the sensillum. The pore openings (arrowheads) on the surface of the sensillum are very small. D: Oblique transverse section of the basal part of the shaft, where the porous part of the cuticle merges in an non-porous part. Note an inflated outer dendritic segment. E: Small dendritic branches and the large inflated dendritic segment come in close contact with the pore tubles. F: Several dendritic branching points (arrowheads) are visible in this section. G: Oblique section of the socket. H: Magnification of the 18 dendritic segments shown in Figure G. Only few, loosely arranged electron-dense remnants of a dendritic sheath are present. I: This further posterior section shows 10 outer dendritic segments embedded in a matrix of dendritic sheath material. J: Four large outer dendritic segments are present below the socket. K: Finally, only two outer dendritic segments represent the entire innervation of the S10 sensillum. Abbr.: Cu, cuticle; dB, dendritic branches; dS, dendritic sheath; oD, outer dendritic segment; Po, pore; pT, pore tubules; RLy, receptor lymph; toC, tormogen cell.

The small, conical S10 sensillum is present once on maxillary and once on labial palps (Figs. 1F, H). The sensillum surface is slightly sculptured (Fig. 9A), but sections reveal the porous character of the shaft (Figs. 9B–E). Many fine dendritic profiles occur in the apical part of the sensillum (Fig. 9B). They get in close contact with pore tubules (Figs. 9C, E). Large, most likely inflated dendritic profiles can be seen in the basal portion of the shaft (Fig. 9D). The fine profiles branch off from these large profiles (Fig. 9F). The sensillum socket comprises 18 outer dendritic segments, joined by loose fibers of a dendritic sheath (Figs. 9 G, H). At deeper section levels the number of dendrites decreases to two and the sheath becomes more and more condensed (Figs. 9 I–K).

**Figure 10:**
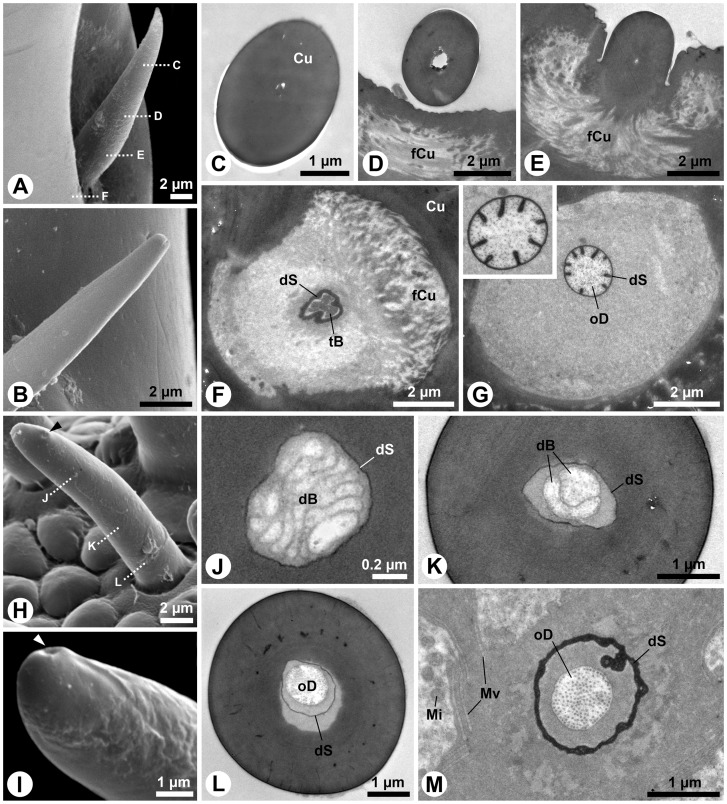
S11 and S12 sensilla of the palps of *M. melolontha* larvae. A–B: SEM. A: S11 sensillum with a pointed tip on a maxillary palp. Dotted lines indicate approximate cutting planes of transverse sections shown in Figures C–F. B: S11 sensillum with a blunt tip from a different maxillary palp. C–G: TEM. C: Oblique section of the sensillum tip. Note the massive cuticle and sparse lumen. D: This section represents the middle portion of the shaft. A lumen is visible, but it is empty. E: Oblique section of the area where the shaft merges in the flexible cuticle of the socket. Note the minute lumen of the shaft. F: A little deeper inside the socket, a thick dendritic sheath with a single tubular body, attached to the flexible cuticle, becomes visible. G: Below the socket only one large ensheathed outer dendritic segment can be found. Note that the number of radial folds of the dendritic sheath changes in different section levels (see inset). H, I: SEM. H: Slightly bent S12 sensillum from the labial palp, bearing a subterminal pore opening (arrowhead). Dotted lines indicate approximate cutting planes of transverse sections shown in Figures J–L. I: The subterminal pore (arrowhead) of this S12 sensillum from a different labial palp opens much closer to the apex (cp. Figure H). J–M: TEM. J: Lamellate dendritic profiles are present in the apical part of the sensillum. K: In this section only two dendritic profiles are visible. L: Shortly above the socket only one dendrite remains inside the dendritic sheath. M: This single dendrite can also be found deeply below the sensillum socket. Abbr.: Cu, cuticle; dB, dendritic branches; dS, dendritic sheath; fCu, flexible cuticle; Mi, mitochondrion; Mv, microvilli; oD, outer dendritic segment; tB, tubular body; tP, terminal pore.

S11 is another small, conical sensillum of the maxillary palps (Fig. 1E). The tip is usually fine (Fig. 10A) but occasionally blunt types are found (Fig. 10B). The shaft lacks any sensory structures (Figs. 10C, D). It merges in a socket with large areas of flexible cuticle (Fig. 10E). A single, large tubular body, surrounded by a thick dendritic sheath, is attached to the flexible cuticle of the socket (Fig. 10F). Below the socket, the corresponding dendritic sheath shows conspicuous radial folds, which divide the periphery of the outer dendritic segment (Fig. 10G) and vary in quantity at different section levels (inset in Fig. 10G).

The S12 sensillum is a single small, slender sensillum, which is exclusively located in the apical sensillum field of the labial palps (Fig. 1H). It is poreless and bears a subterminal (Fig. 10H) or terminal pore (Fig. 10I). The lumen contains lamellated dendritic branches surrounded by a thin sheath (Fig. 10J). Further basally, only two dendritic branches are visible (Fig. 10K). The sensillum is innervated by one ensheathed outer dendritic segment, which enters the shaft before it starts to lamellate (Figs. 10L, M).

### Electroantennograms (EAG) and Electropalpograms (EPG)

**Figure 11:**
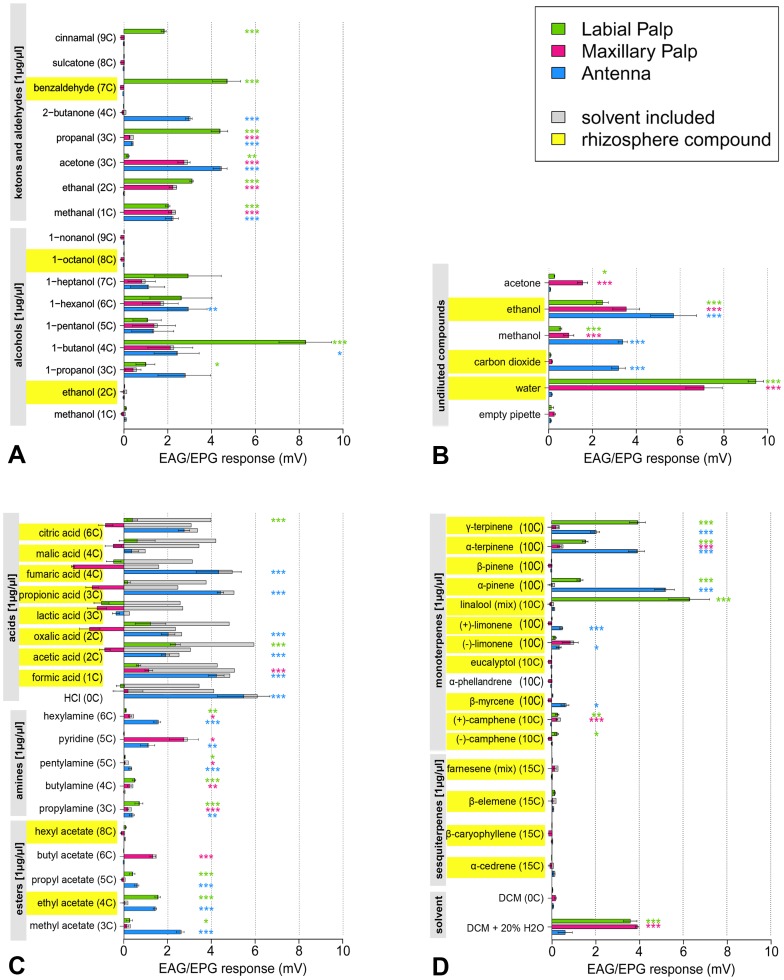
Mean EAG and EPG amplitudes for recordings on antennae (**blue bars**)**, maxillary** (**pink bars**) **and labial palps** (**green bars**) **from third instar** ***M. melolontha*** **larvae whole-body mounts** (**n = 15 replicates on 6 animals** (**1–3 per animal**))**.** Response to respective controls (empty pipette, DCM, dist. water and DCM supplemented by 20% water) has been subtracted. The grey bars behind colored bars display gross responses without solvent correction. Asterisks indicate significantly higher responses to the tested compound than to respective solvents (Welch two sample t-tests with sqrt transformed data). Significance levels: *** at p<0.001; ** at p<0.01 and * at p<0.05. A: alcohols, ketones and aldehydes at a concentration of 1µg/µl in DCM. B: Undiluted compounds, stimulation with empty pipette and CO_2_, C: Amines, esters at a concentration of 1µg/µl in DCM and acids at the same concentration in DCM supplemented by 20% dist. water. D: Monoterpenes, sesquiterpenes and solvents at a concentration of 1µg/µl in DCM.

Electrophysiological recordings are conducted on 3^rd^ instar *M. melolontha* larvae antennae (EAG), maxillary and labial palps (EPG). The mean responses to tested compounds range from 0.03mV±0.01mV (solvent DCM) to 8.81±0.86mV (water) in labial palps, 0.11±0.02mV (empty pipette) to 6.89±1.7mV (water) in maxillary palps, and 0.06mV±0.016mV (solvent DCM) to 5.7±1.05mV (ethanol) in antennae. Overall, significant responses were found for compounds from all tested chemical classes, i.e. alcohols, aldehydes, ketones (Fig. 11A), CO_2_ and water (Fig. 11B), acids, amines, esters (Fig. 11C) and terpenoids (Fig. 11D). However, none of the head appendages respond to the tested sesquiterpenes β-elemene, β-caryophyllene, α-cedrene, and farnesene isomeres. In contrast, all appendages respond to propanal, acetone, methanal, propyl- butyl- and hexylamine, and α-terpinene. Both palps respond to changes in humidity, to butylamine and ethanal. Antennae and labial palps both respond to 1-butanol, 1-propanol, citric and acetic acid, methyl ethyl and propyl acetate, γ-terpinene and α-pinene. Interestingly, β-pinene elicits no response on these appendages. Moreover, (+)-camphene and α-terpinene elicit responses in maxillary palps, whereas no significant response is observed to (−)-camphene and γ-terpinene. This observation indicates enantio- and isomer-specific perception of these compounds. Other than antennae and maxillary palps, the labial palps respond significantly to cinnamaldehyde, benzaldehyde, linalool and (−)-camphene. Responses to CO_2_ (4%), 2-butanone, 1-hexanol, fumaric, propionic, oxalic and hydrochloric acid, (±)-limonene and β-myrcene are restricted to the antennae.

Butyl acetate is the only tested component eliciting responses exclusively in the maxillary palps, but not coevally on antennae or labial palps.

## Discussion

Our ultrastructural and electrophysiological studies reveal highly developed chemosensory structures in soil-dwelling *M. melolontha* larvae. Olfactory, as well as contact-chemosensory neurons, are present in sensilla on antennae, maxillary and labial palps. Morphological characteristics indicate olfactory function in three out of 17 sensillum types located on larval antennae and palps olfactory, and gustatory function for seven sensillum types. A multitude of host-derived compounds elicit physiological responses in antennae and palps. Each head appendage has its own olfactory response profile. Some responses are appendage-specific down to the level of enantiomers (Fig. 11D).

The pore plates on the larval antenna are the most prominent chemosensory structures, both in terms of area covered as well as numbers of innervating sensory neurons. The apices of all examined head appendages are dominated by contact chemo-sensilla or multimodal mechano- and contact chemo-sensilla equipped with single terminal pores and distinct dendritic structures. The most abundant peg-like sensillum type S2, a combined contact chemo- and mechano-sensillum, occurs on antennae, maxillary and labial palps. Further contact-chemoreceptive sensilla are S3, S4, S5, S8 and S9.

Larvae of *M. melolontha* have been observed pushing their heads into the sidewalls of their burrows ([41] and personal observations), which is interpreted as probing behavior with antennal and palpal apices (Fig. 1A, B) predominantly tasting the surrounding matrix. Hence, the corresponding sensilla may serve to orient along gradients of water-soluble chemicals present on the matrix. In contrast, size (S7, S10) or position (S7, pore plates) of the olfactory sensilla prevent direct contact to the substrate and thus warrant stimulation through the gas phase only. Behavior and spatial arrangement of sensilla indicate that the larvae use both contact and olfactory cues present in the rhizosphere.

### Sensillum characterization and terminology

Following Keil [42] the olfactory sensilla on *M. melolontha* larval head appendages are single walled sensilla basiconica, i.e. tapering pegs with wall pores (S7, S10), and sensilla placodea (pore plates). All contact chemo-sensilla fall into different sub-categories of single walled sensilla chaetica with a pore at or close to the tip (S3, S4, S5, S8, S9). Interestingly, none of the observed sensilla displays a double cuticular wall, and all sensilla with mechano-sensory function except S1, S13 and S14 fall into the category of s. chaetica as well. Despite its untypical furcate tip, S1 appears to be a mechanosensory sensillum trichodeum. The function of the furcation (Fig. 1D, 5A), however, remains elusive.

### Olfactory sensilla – multiporous single walled

Antennal pore plates are common in scarab larvae. Their abundance on the apical antennal segment may differ from one [43] to more than a dozen [44], [45] in xylophagous and saprophagous larvae [46], but there are always three in rhizophagous larvae, irrespective of subfamily affiliation ([19], , and this study). The presence of minute pores with pore tubules and subjacent branching outer dendritic segments indicate their olfactory function. Some adult scarab beetles bear small but ‘larval-like’ planar sensilla placoidea [17], while in other species these organs are superficially modified to dome-shaped [49] or sculptured s. placoidea with foldings or cavities [18]. The innervation pattern of adult s. placoidea, however, is in each case similar to the sensory units we found underneath the cuticle of larval pore plates (cp. Review by [50] and citations therein). We therefore interpret the functional sensory units underneath the pore plates as cryptic s. placodea, homologous to the adult s. placodea, and the pore plates as multi-sensillum olfactory fields. Based on the average size of the pore plates in relation to the average distance between adjacent dendritic bundles, we estimate a number of 80–120 sensory units in each of the three large pore plates on the distal antennal segment and about 10–15 units for the small pore plate on the cuticular protrusion of the subapical antennal segment. Hence, about 300 sensory units with a total number of about 1000 sensory neurons innervate the pore plates of one larval antenna (Fig. 3). Regarding the number of functional sensilla and olfactory sensory neurons (OSNs), *M. melolontha* larvae thus resemble adult insects like *Drosophila melanogaster* [51].

Only one olfactory basiconic sensillum, innervated by a maximum of two or three OSNs is located on the tip of each palp (S10), and on the cuticular protrusion of the subapical antennal segment (S7), respectively (Table 1, Fig. 7 & 9). This clearly indicates that major olfactory input comes from the multi-sensillum olfactory fields on the antennae.

### Contact chemo-sensilla – single terminal pores

The number of outer dendritic segments indicates 4 or 5 chemoreceptive neurons for most contact chemo-sensilla, except for S9a & b with 6 chemoreceptive neurons per sensillum. In contrast to sugar sensitive cells, which are commonly found in insects, pH sensitive cells have to our knowledge so far only been described in ground beetles [52]. In a set of preliminary experiments we observed behavioral responses to diverse sugars and organic acids (Eilers, unpubl.). We therefore assume that sugar and pH-sensitive neurons are present in the s. chaetica. Single gustatory sensillum recordings were attempted to identify the responsive profiles of the s. chaetica. However, well established protocols (e.g. [53], [54] did not result in successful stimulation of taste sensilla on the palps of *M. melolontha*. The lack of response to all applied gustatory stimuli (sugars, salts, organic acids, caffeine, and aqueous dandelion root extracts) may be related to a missing fulfillment of essential homeostatic needs in the larvae, as the experiments were not performed in their natural environment, soil. External signals, which might have interfered with the gustatory recordings, are for instance the presence of light, inadequate moisture, temperature, oxygen or carbon dioxide levels, or – despite all experimental efforts – the presence of vibrations or similar mechanical disruption. An insects homeostatic sensory system operates in a narrow range and even a minor discrepancy from the preferred milieu may induce major physiological changes in the animal [55], [56].

### Hygro- and thermoreception

Avoiding heat, drought and excess wetness is crucial for the survival of *M. melolontha* larvae [41], [57]. Only maxillary and labial palps of *M. melolontha* larvae respond to changes in air humidity in our electrophysiological experiments. Highly lamellated dendritic structures as found in the digitiform organ on the maxillary and sensillum S11 on the labial palps, are characteristic for thermo-hygroreceptors [58]. We therefore suggest that the digitiform organ and S11 sensillum are the responsible hygro-/thermoreceptive organs.

### Electrophysiological responses to volatile stimuli

Out of the 52 compounds, relevant for below ground living insects or analogs of these compounds, the antenna of *M. melolontha* larvae respond to 27, the maxillary palp to 13 and the labial palp to 23 compounds. Sixteen of the tested compounds elicit similar responses in antennae and labial palps. All classes of tested volatiles aside from sesquiterpenoids elicit antennal responses, among them monoterpenes and 1-hexanol, typical plant volatiles. The antennal s. placodea most probably have an important role in the detection of these typical plant derived compounds (but see below). Furthermore, the antennae are the only head appendages responding to CO_2_. Cockchafer larvae were shown to orient upwards in faint gradients of 0.001 vol%/cm within a wide range of ambient CO_2_ concentrations [26]. Together, sensitive behavioral and robust electrophysiological responses indicate that rather multiple than a single or few neurons mediate responses to CO_2_. Similar to CO_2_, 2-butanone elicits electrophysiological responses on the antennae only. This compound activates CO_2_ receptive OSNs also in mosquitoes [59], [60], [61]. Taken together with our results this indicates that the s. placodea on the antennae are involved in CO_2_ perception. Considering that CO_2_ may be present as carbonic acid in moist soil, further possible candidates for larval CO_2_ detection would be contact chemoreceptors present only on the antennae, such as S4, and S5.

Different response profiles are characteristic to OSNs housed within single sensilla like the cryptic s. placodea found in *M. melolontha* larvae [62], [63]. CO_2_-sensitive neurons may pair with other OSNs [64]. Interactions between CO_2_ and other rhizosphere compounds have been demonstrated at the behavioral level [27]. Whether this is indeed reflected in co-localized OSNs for odorants and CO_2_ requires single sensillum recordings for confirmation.

Exclusively labial palps respond to benzaldehyde and cinnamaldehyde, typical aromatic plant volatiles eliciting responses in antennae of a wide array of adult insects (e.g. [65], [66], [67], [68], [69]). Butyl acetate, for instance, elicits a response in maxillary palps only, while methyl, ethyl and propyl acetate elicit responses in labial palps and antennae only. Hexylamine and 1-hexanol elicit responses in antennae, while no antennal response is detected to hexyl acetate (all C6). Similarly, butyl acetate and butylamine elicit no responses in antennae, but 1-butanol does (all C4). Some responses are even head appendage-specific when comparing enantiomers. The labial palps respond to (−)-camphene, while maxillary and labial palps respond to (+)- camphene. Antennae respond to most of the tested organic acids, labial palps respond to citric and acetic acid and maxillary palps to stimulation with formic acid (Fig. 11C), although stimulated with gas phase. Thus, EAG and EPG responses cannot be assigned to chemical classes or carbon chain lengths (volatility), but are head appendix specific at an individual compound base.

Following morphologic criteria, each palp bears only two OSNs. It is unlikely that electroantennographic or –palpographic signals are picked up from single neurons. Despite the prominent olfactory pore plates on the antennae this reasoning together with the wide variety of appendage-specific responses rather indicate that (i) there is no clear-cut distinction between antennae and palps with respect to olfactory function and that (ii) typical gustatory sensilla most probably have a dual function serving both olfaction and taste. Four-to-six sensory neurons are present in each s. chaeticum, a sufficient number to allow for a set of taste neurons to be combined with OSNs within one sensillum. In larvae of the sphingid hawk moth *Manduca sexta* thick walled gustatory sensilla on maxillary palps were shown to have olfactory capabilities as well. They respond to plant derived volatile substances besides their response to salt and sugar [70]. Again, single sensillum recordings are required to corroborate our hypothesis in *M. melolontha*. Whether the respective sensory neurons project into the suboesophagial ganglion, the primary center for processing of gustatory information [71] or the antennal lobe, the primary center for processing of olfactory input [72] also remains to be determined.

Our findings clearly show that *M. melolontha* larvae possess intriguingly well developed chemosensory organs equivalent to those of many adult insects. In this issue of PLoS One >Weissteiner et al.< (citation will be adapted upon acceptance) report that the antennal lobe, the first brain center to process olfactory input, is composed of about 70 glomeruli in the congeneric *M. hippocastani*. The number of glomeruli is indicative of the diversity of olfactory receptor proteins and thereby of OSN types [73], and corresponds well to what has been found in adult model insects for olfactory research [74], [75]. Scarab beetles spend the majority of their lifecycle as larvae below ground, feeding on plant roots. The developmental period, in which host location in a complex matrix is a major task, may have favored the evolution of a larval chemosensory equipment comparable to adult insects.

## Supporting Information

Table S1 **Sensory organs on antennae** (**A**)**, galea** (**G**)**, maxillary** (**M**) **and labial palps** (**L**) **of species belonging to different Coleopteran and Lepidopteran families and subfamilies.** Abbreviations: #?, unknown number; A, Antenna; ap, apical; BC, basiconic; CF, campaniform; CH, chaetica; CP, present in cuticular protrusion on postapical antennal segment; CR, chemoreceptor; di, distal; Do, digitiform organ; dor, dorsal; Fo, foliphagous; G, Galea; GR, contact-chemoreceptor (gustatory); Her, herbivorous (foliage, blossoms, seeds or stem); HR, hygroreceptor; L, labial palps; lat, lateral; LM, light or sterio microscopy; M, maxillary palps; MR, mechanoreceptor; NP, aporous; OR, olfactory receptor; PP, sensory pore plate; Pred, predatory; Rhz, rhizophagous; Sa, saprophagous/ detritus feeder; SC, styloconic; Sca, scavenger; SEM, scanning electron microscopy; TEM, transmission electron microscopy; TR, thermoreceptor; Xy, xylophagous or saproxylophagous; UP, uniporous; ven, ventral; WP, wall pores/multiporous.
(DOC)
