# Supplementary material for: Sensing the Underground – Ultrastructure and Function of Sensory Organs in Root-Feeding Melolontha melolontha (Coleoptera: Scarabaeinae) Larvae
Source: PLoS One. 2012 Jul 25;7(7):e41357. doi: 10.1371/journal.pone.0041357 (PMC3405142; doi:10.1371/journal.pone.0041357)
Supplement: Table S1 — Sensory organs on antennae (A), galea (G), maxillary (M) and labial palps (L) of species belonging to different Coleopteran and Lepidopteran families and subfamilies. Abbreviations: #?, unknown number; A, Antenna; ap, apical; BC, basiconic; CF, campaniform; CH, chaetica; CP, present in cuticular protrusion on postapical antennal segment; CR, chemoreceptor; di, distal; Do, digitiform organ; dor, dorsal; Fo, foliphagous; G, Galea; GR, contact-chemoreceptor (gustatory); Her, herbivorous (foliage, blossoms, seeds or stem); HR, hygroreceptor; L, labial palps; lat, lateral; LM, light or sterio microscopy; M, maxillary palps; MR, mechanoreceptor; NP, aporous; OR, olfactory receptor; PP, sensory pore plate; Pred, predatory; Rhz, rhizophagous; Sa, saprophagous/ detritus feeder; SC, styloconic; Sca, scavenger; SEM, scanning electron microscopy; TEM, transmission electron microscopy; TR, thermoreceptor; Xy, xylophagous or saproxylophagous; UP, uniporous; ven, ventral; WP, wall pores/multiporous. (DOC) [file pone.0041357.s001.doc]

| **Affiliation** | **Develop. stage** | **Structure** | **Location,**  **abundance** | **Species** | **Method** | **Hypothe-sized function** | **Diet** | **Origin** | **Reference** |
| --- | --- | --- | --- | --- | --- | --- | --- | --- | --- |
| **Coleoptera:**  **Carabidae** | **3^rd^ instar**  **(final)** | **PP** | **A:** 1CP | *Ophonus ardosiacus* L. | SEM, TEM | OR | Her | Italy | [1] |
|  | **2^nd^ instar** |  | not present | *Pterostichus melanarius* I. | SEM | / | Pred  (slugs) | Great Britain | [2] |
|  |  | **Setae** | **A:**  4 trichoid,  4 basiconic |  |  | n.a. |  |  |  |
|  |  | **Do** | not present |  |  | / |  |  |  |
|  | **1^st^ to 3^rd^ instar** |  | **M:** 1-28 (UP)  **L:** 0-50 (UP) | 22 species in 16 genera belonging to 10 tribes | SEM,  TEM | MR, CR or HR | n.a. | Italy, Slovenia | [3] |
|  |  | **Setae** | **M:** 1-12  **L:** 7-61 |  |  | n.a. |  |  |  |
|  |  |  |  |  |  |  |  |  |  |
| **Coleoptera:**  **Chrysomelidae** | **Adult** | **PP** | not present | *Phyllotreta cruciferae* G., *Psylloides punctulata* M.,  *Epitrix cucumeris* H.,  *Psylloides affinis* P. | SEM | / | Her | Canada | [4] |
|  |  | **Setae** | **A:** >200 |  |  | n.a. |  |  |  |
|  | **Larva, unknown instar** |  | **G:** >2 | *Entomosceiis americana* B. | SEM, TEM | MR and GR |  | USA | [5] |
|  | **3^rd^ instar**  **(final)** |  | **G:** >2 | *Leptinotarsa decemlineata* S. |  | n.a. |  | Canada | [6] |
|  |  | **Setae** | **M:** 16  **L:** 11 |  |  | GR |  | Moscow | [7] |
|  |  | **Do** | **M:** 1 |  |  | MR |  |  |  |
|  |  |  |  |  |  |  |  |  |  |
| **Coleoptera:**  **Curculionidae** | **3^rd^ instar**  **(final)** | **Do** | **M:** 1 | *Ceutoryhnchus obstrictus* M. | SEM | n.a. | Her | Europe, North America | [8] |
|  |  | **Setae** | **A:** 6  **M:** 12 |  |  |  |  |  |  |
|  |  | **PP** | **A:** 1 |  |  |  |  |  |  |
| **Coleoptera:**  **Dermestidae** | **Adult** | **Do** | **M:** 11 | *Dermestes maeulatus* D.G. | SEM, TEM | TR/HR or CO_2_ | Sca | Germany | [9] |
| **Coleoptera:**  **Dynastidae** | **Adult** | **PP** | **A:** >45,000 | *Oryctes rhinoceros* L. | SEM, TEM | OR | Her | Indonesia | [10] |
|  |  |  |  |  |  |  |  |  |  |
| **Coleoptera:**  **Dytiscidae** | **Adult** | **Do** | **M:** 11 | *Agabus bipustulatus* L. | SEM, TEM | TR | Her? Aquatic | n.a. | [11] |
|  |  |  |  |  |  |  |  |  |  |
| **Coleoptera:**  **Elateridae** | **Larva, unknown instar** | **Do** | **M:** 1 | *Agriotes lineatus obscurus* L. *Limonius californicus* M. | SEM | n.a. | n.a. | Canada | [12] |
|  |  | **Setae** | **M:** ca. 60  **L:** ca. 14 |  |  |  |  |  |  |
|  | **Final instar** | **PP** | **A:** 1ven, 1dor, 1CP | *Aeolus cinctus* C. | LM? |  | Pred (termites) | Brazil | [13] |
|  |  | **Do** | **M:** 1 |  |  |  |  |  |  |
|  |  |  |  |  |  |  |  |  |  |
| **Coleoptera:**  **Hydrophilidae** | **Adult** | **Do** | **M:** 4-6 | *Hydrobius fuscipes* L. | SEM,  TEM | TR | Her? Aquatic | n.a. | [11] |
|  |  |  |  |  |  |  |  |  |  |
| **Coleoptera:**  **Scarabaeidae:**  **Aphodiinae** | **3^rd^ instar**  **(final)** | **PP** | **A:** 1dor,1ven | *Ataenius opatrinus H.,*  *Ataenius picinus H.,*  *Ataenius simulator* H.,  *Ataenius. platensis* B.,  *Ataenius strigicauda* B. | LM | n.a. | Sa, Rhz | Uruguay | [14] |
| **Cetoniinae** |  |  | **A:** 3di, 2lat | *Ichnestoma stobbiai* H. |  |  | Sa | South Africa | [15] |
|  |  |  | **A:** 10-11 | *Heterorrhina smaragdin* V. |  |  | Sa, Xy | Nigeria | [16] |
|  |  |  | **A:** 13-17 | *Platygenia barbata* A. |  |  |  |  |  |
|  |  |  | **A:** 7-8 | *Gnathocera trivittata* S. |  |  |  |  |  |
|  |  |  | **A:** 4 | *Grammopyga cincticollis* H., *Clastocnemis quadrimaculatus* A. |  |  |  |  |  |
|  |  |  | **A:** 5 | *Pachnoda marginella* F. |  |  |  |  |  |
|  |  |  | **A:** 2ven, 3dor, 1CP | *Coelocorynus desfontainei* A., and *C. opacicauda* A. |  |  |  | Cameroon | [17] |
| **Coleoptera:**  **Scarabaeidae:**  **Cetoniinae** | **3^rd^ instar**  **(final)** | **PP** | **A:** 3dor,3ven | *Hoplopyga singularis* G.P.,  *Hologymnetis cinerea* G.P. | LM | n.a. | Sa | Brazil, Mexico | [18] |
|  |  |  | **A:** 4dor | *Tropinota squalida* S. |  |  |  | Spain |  |
|  |  |  | **A:** 4dor, 1CP | *Oxythyrea funesta* P. |  |  |  |  |  |
|  |  |  | **A:** 4dor | *Aethiessa floralis* F. |  |  |  |  |  |
| **Dynastinae** |  |  | **A:** 7dor, ?#ven, 1CP | *Oryctes nasicornis* L. |  |  | Sa, Xy | Russia | [19] |
|  |  |  | **A:** 4-5 | *Megasoma sleeperi* H |  |  | Xy | USA | [20] |
|  |  |  | **A:** 12dor, 15ven | *Strategus syphax* F. |  |  | unknown | Caribbean | [21] |
| **Melolonthinae** |  |  | **A:** 3, 1CP | *Serica brunnea* L. |  |  | Rhz | Great Britain | [22] |
|  |  | **Setae** | **A:** CP: 4 |  |  |  |  |  |  |
| **Orphninae** |  | **PP** | **A:** 1dor | *Aegidium cribratum* B. |  |  | Sa, Xy | Mexico | [23] |
| **Rutelinae** |  |  | **A:** 1dor,2ven | *Anisoplia baetica* E.,  *Anisoplia depressa* E., *Anisoplia remota* R.,  *Anthoplia floricola F.* |  |  | Rhz | Europe, Africa | [24] |
|  |  |  | **A:** 2 dor | *Paraheterosternus luedeckei* B. |  |  | Xy | Mexico | [25] |
|  |  |  | **A:** 6dor,7ven | *Chasmodia collaris* B. |  |  |  | USA | [26] |
|  |  |  | **A:** 4dor,3ven | *Chasmodia cincticollis* B. |  |  |  |  |  |
|  |  | **Setae** | **A:** 3-4  **M:** approx. 20  **L:** approx. 13 | *Phyllopertha horticola* | SEM, TEM |  | Rhz | Europe, Asia | [27] |
|  |  | **PP** | **A:** >2 |  |  | OR |  |  |  |
|  | **Adult** |  | **A:** 200 | *Popillia japonica* N. |  |  | Fo | Japan | [28] |
| **Coleoptera:**  **Scarabaeidae:**  **Scarabaeinae** | **3^rd^ instar**  **(final)** | **Setae** | **A:** 8 | *Cotinis nitida* | SEM | n.a. | n.a. | n.a. | [29] |
|  |  |  |  |  |  |  |  |  |  |
| **Coleoptera:**  **Tenebrionidae** | **Final**  **instars** | **Setae** | **A:** 9 | *Tribolium confusum*  *Tribolium castaneum* | SEM,  TEM | n.a. | Her (flour) | n.a. | [30] |
|  |  | **PP** | **A:** 1 |  |  |  |  |  |  |
|  | **Adult** | **Do** | **M:** 14 | *Tenebrio molitor* L. |  | TR/HR- or CO_2_ | Her (flour), Pred | Germany | [9] |
|  |  |  |  |  |  |  |  |  |  |
| **Lepidoptera:**  **Lymantriidae** | **5^th^ instar**  **(final)** | **Setae** | **M:** 8  **G:** 2 UP,  3 NP | *Lymantria dispar* L. | SEM, TEM | n.a. | Her | USA | [31] |
|  |  |  |  |  |  |  |  |  |  |
| **Lepidoptera:**  **Noctuidae** | **5^th^ instar**  **(final)** | **Setae** | **M:** 7  **L:** 2 | *Heliothis virescens* F | SEM | n.a. | Her | n.a. | [32] |
|  |  | **PP** | **M:** 2 |  |  |  |  |  |  |
|  |  | **Do** | **M:** 1 |  |  |  |  |  |  |
|  |  | **Setae** | **M:** 9  **L:** 2 | *Heliothis zea* B |  |  |  |  |  |
|  |  | **PP** | **M:** 2 |  |  |  |  |  |  |
|  |  | **Do** | **M:** 1 |  |  |  |  |  |  |
|  |  | **Setae** | **G:**  2 SC UP,  3 BC WP | *Spodoptera frugiperda* S.*, Choristoneura fumiferana* C., *Lymantria dispar* L.,  *Prunus virginiana* L. | SEM,  TEM | SC: GR  BC: OR |  | Canada | [33] |
|  |  |  | **G:**  2 SC UP,  3 BC WP,  1 CF UP | *Malacosoma lutescens* N.D., *Trichoplusia ni* H.,  *Mamestra configurata* W., |  | SC: GR  BC: OR |  |  |  |
|  |  |  | **M:** 8 UP  **L:** 2 NP | *Euxoa messoria* H. |  | UP: CR  NP: MP | n.a. | n.a. | [34] |
| **Lepidoptera:**  **Noctuidae** | **5^th^ instar**  **(final)** | **Do** | **M:** 1 | *Euxoa messoria* H. | SEM, TEM | MR | n.a. | n.a. | [34] |
|  |  | **PP** | **M:** 3 |  |  | CR |  |  |  |
|  | **3^rd^ to 5^th^ instar** |  | **M:** 2 | *Helicoverpa armigera* H. |  | CO_2_ |  |  | [35] |
|  |  | **Do** | **M:** 1 |  |  | TR, CO_2_ |  |  |  |
|  |  | **Setae** | **M:**  3 BC WP  5 BC UP  1 CF NP |  |  | WP: OR  UP: GR  NP: MR |  |  |  |
|  |  |  |  |  |  |  |  |  |  |
| **Lepidoptera:**  **Pyralidae** | **4^th^ instar** | **Setae** | **A:**  2 CH NP,  1 SC NP,  3 BC WP  **M:**  8 BC UP  **L:**  1 CH NP  1 SC NP  **G:**  2 SC UP  2 BC NP  1 CP  3 CH NP | *Homoeosoma nebulella* D.S. | SEM | WP: OR  NP: TR | Her | France | [36] |
|  |  | **Do** | **M:** 1 |  |  | TR |  |  |  |
|  |  | **PP** | **M:** 1 |  |  | n.a. |  |  |  |
|  |  |  |  |  |  |  |  |  |  |
| **Lepidoptera:**  **Yponomeutidae** | **5^th^ instar (final)** | **Do** | **M:** 1 | *Yponomeuta cagnagellus* | SEM | n.a. | Her | Nether-lands | [37] |
|  |  | **Setae** | **M:** 1 |  |  |  |  |  |  |

**References:**

1. Giglio A, Brandmayr P, Ferrero EA, Giulianini PG, Perrotta E, et al. (2008) Ultrastructure of the antennal sensorial appendage of larvae of *Ophonus ardosiacus* (Lutshnik, 1922) (Coleoptera, Carabidae) and possible correlations between size and shape and the larval feeding habits. Zoologischer Anzeiger 247: 209-221.

2. Thomas RS, Glen DM, Symondson WOC (2008) Prey detection through olfaction by the soil-dwelling larvae of the carabid predator *Pterostichus melanarius*. Soil Biology & Biochemistry 40: 207-216.

3. Giglio A, Ferrero EA, Perrotta E, Tripepi S, Brandmayr TZ (2003) Ultrastructure and comparative morphology of mouth-part sensilla in ground beetle larvae (Insecta, Coleoptera, Carabidae). Zoologischer Anzeiger 242: 277-292.

4. Ritcey GM, McIver SB (1990) External Morphology of antennal sensilla of four species of adult flea beetles (Coleoptera: Chrysomelidae: Alticinae). International Journal of Insect Morphology & Embryology 19: 141-153.

5. Mitchell BK, Whitehead AT, Backus E (1979) Ultrastructure of the Lateral and Medial Galeal Sensilla of the Larva of the Red Turnip Beetle, *Entomoscelis Americana* Brown (Coleoptera, Chrysomelidae). International Journal of Insect Morphology & Embryology 8: 289-295.

6. Sen A, Mitchell BK (1987) Ultrastructure of the galeal sensory complex in adults of the Colorado potato beetle, *Leptinotarsa decemlineata*. Physiological Entomology 12: 81-90.

7. Farazmand H, Chaika SY (2008) Morphology and ultrastructure of chemosensory sensilla of labio-maxillary complex in the Colorado potato beetle, *Leptinotarsa decemlineata* (Col.: Chrysomelidae), larvae. Journal of Entomological Society of Iran 27: 1-11.

8. Dosdall LM, McFarlane MA (2004) Morphology of the pre-imaginal life stages of the cabbage seedpod weevil, *Ceutorhynchus obstrictus* (Marsham) (Coleoptera : Curculionidae). The Coleopterists Bulletin 58: 45-52.

9. Honomichl K, Guse G-W (1981) Digitiform sensilla on the maxillar palp of Coleoptera. 3. Fine-Structure in *Tenebrio molitor* L. and *Dermestes maculatus* Degeer. Acta Zoologica 62: 17-25.

10. Renou M, Tauban D, Morin JP (1998) Structure and function of antennal pore plate sensilla of *Oryctes rhinoceros* (L.) (Coleoptera : Dynastidae). International Journal of Insect Morphology & Embryology 27: 227-233.

11. Guse G-W, Honomichl K (1980) Die digitiformen Sensillen auf dem Maxillarpalpus von Coleoptera II. Feinstruktur bei *Agabus bipustulatus* (L.) und *Hydrobius fuscipes* (L.). Protoplasma 103: 55-68.

12. Doane JF, Klingler J (1978) Location of CO_2_-receptive sensilla on larvae of wireworms *Agriotes lineatus-obscurus* and *Limonius californicus*. Annals of the Entomological Society of America 71: 357-363.

13. Casari SA (2006) Larva, pupa and adult of *Aeolus cinctus* Candeze (Coleoptera, Elateridae, Agrypninae). Revista Brasileira De Entomologia 50: 347-351.

14. Verdu JR, Galante E (1999) Larvae of *Ataenius* (Coleoptera : Scarabaeidae : Aphodiinae): generic characteristics and species descriptions. European Journal of Entomology 96: 57-68.

15. Deschodt CM, Scholtz CH, Kryger U (2009) Description of the larva and pupa of *Ichnestoma stobbiai* Holm 1992 (Scarabaeidae: Cetoniinae), a range-restricted species of conservation concern. African Entomology 17: 43-50.

16. Jerath ML, Unny KL (1965) Larvae of six genera of Cetoniinae from Eastern Nigeria (Coleoptera: Scarabaeidae). The Coleopterists Bulletin 19: 59-64.

17. Sipek P, Gill BD, Grebennikov VV (2009) *Afromontane Coelocorynus* (Coleoptera: Scarabaeidae: Cetoniinae): Larval descriptions, biological notes and phylogenetic placement. European Journal of Entomology 106: 95-106.

18. Micó E, Hall WE, Ratcliffe BC (2001) Descriptions of the larvae of *Hoplopyga singularis* (Gory and Percheron) and *Hologymnetis cinerea* (Gory and Percheron) with a revised key to the larvae of New World Gymnetini (Coleoptera : Scarabaeidae : Cetoniinae). The Coleopterists Bulletin 55: 205-217.

19. Grebennikov VV, Scholtz CH (2004) The basal phylogeny of Scarabaeoidea (Insecta : Coleoptera) inferred from larval morphology. Invertebrate Systematics 18: 321-348.

20. Van Dam M, Van Dam A, Wilcox MD (2006) Description of the third-instar larva and adult male of *Megasoma sleeperi* Hardy (Scarabaeidae : Dynastinae). The Coleopterists Bulletin 60: 59-67.

21. Ratcliffe BC, Chalumeau F (1980) *Strategus syphax* (Fabr.): A description of the third instar larva and pupa (Coleoptera: Scarabaeidae: Dynastinae). The Coleopterists Bulletin 34: 85-93.

22. Jepson WF (1937) Observations on the morphology and bionomics of *Serica brunnea*, L., with notes on allied chafer pests. Part I. The morphology of the larva of *Serica brunnea*, L. Bulletin of Entomological Research 28: 149-165.

23. Moron MA (1991) Larva and pupa of *Aegidium cribratum* Bates (Coleoptera, Scarabaeidae, Orphninae). The Coleopterists Bulletin 45: 360-367.

24. Micó E, Verdú JR, Galante E (2001) Larval morphology of some Anisopliini grain beetles with a key to their larvae (Coleoptera: Scarabaeoidea: Rutelidae: Anomalinae). European Journal of Entomology 98: 311-320.

25. Moron MA, Nogueira G (2000) Third stage larva and pupa of *Paraheterosternus luedeckei* (Becker) (Coleoptera : Melolonthidae; Rutelinae). Journal of the Kansas Entomological Society 73: 62-67.

26. Jameson ML, Moron MA (2001) Descriptions of the larvae of *Chlorota cincticollis* (Blanchard) and *Chasmodia collaris* (Blanchard) (Scarabaeidae:Rutelinae:Rutelini) with a key to the larvae of the American genera of Rutelini. The Coleopterists Bulletin 55: 385-396.

27. Alekseev MA, Sinitsina EE; Chaika, SY (2006) Sensory organs of the antennae and mouthparts of beetle larvae (Coleoptera). Entomological Review 86: 638-648.

28. Kim JY, Leal WS (2000) Ultrastructure of pheromone-detecting sensillum placodeum of the Japanese beetle, *Popillia japonica* Newmann (Coleoptera : Scarabaeidae). Arthropod Structure & Development 29: 121-128.

29. Baker GT, Monroe WA (2005) Sensilla on the adult and larval antennae of *Cotinis nitida* (Coleoptera: Scarabaeidae). Microscopy and Microanalysis 11: 170-171.

30. Behan M, Ryan MF (1978) Ultrastructure of antennal sensory receptors of *Tribolium* larvae (Coleoptera Tenebrionidae). International Journal of Insect Morphology & Embryology 7: 221-236.

31. Shields VDC (2009) Fine structure of the galeal styloconic sensilla of larval *Lymantria dispar* (Lepidoptera: Lymantriidae). Annals of the Entomological Society of America 102: 1116-1125.

32. Baker GT, Parrott WL, Jenkins JN (1986) Sensory receptors on the larval maxillae and labia of *Heliothis zea* (Boddie) and *Heliothis virescens* (F) (Lepidoptera, Noctuidae). International Journal of Insect Morphology & Embryology 15: 227-232.

33. Shields VDC (1996) Comparative external ultrastructure and diffusion pathways in styloconic sensilla on the maxillary galea of larval *Mamestra configurata* (Walker) (Lepidoptera: Noctuidae) and five other species. Journal of Morphology 228: 89-105.

34. Devitt BD, Smith JJB (1982) Morphology and fine structure of mouthpart sensilla in the dark-sided cutworm *Euxoa messoria* (Harris) (Lepidoptera, Noctuidae). International Journal of Insect Morphology & Embryology 11: 255-270.

35. Keil TA (1996) Sensilla on the maxillary palps of *Helicoverpa armigera* caterpillars: in search of the CO_2_-receptor. Tissue & Cell 28: 703-717.

36. Faucheux MJ (1995) Sensilla on the larval antennae and mouthparts of the European sunflower moth, *Homoeosoma Nebulella* Den-and-Schiff (Lepidoptera, Pyralidae). International Journal of Insect Morphology & Embryology 24: 391-403.

37. Roessingh P, Xu S, Menken SBJ (2007) Olfactory receptors on the maxillary palps of small ermine moth larvae: evolutionary history of benzaldehyde sensitivity. Journal of Comparative Physiology a-Neuroethology Sensory Neural and Behavioral Physiology 193: 635-647.
